# Cohesin Proteins Promote Ribosomal RNA Production and Protein Translation in Yeast and Human Cells

**DOI:** 10.1371/journal.pgen.1002749

**Published:** 2012-06-14

**Authors:** Tania Bose, Kenneth K. Lee, Shuai Lu, Baoshan Xu, Bethany Harris, Brian Slaughter, Jay Unruh, Alexander Garrett, William McDowell, Andrew Box, Hua Li, Allison Peak, Sree Ramachandran, Chris Seidel, Jennifer L. Gerton

**Affiliations:** 1 Stowers Institute for Medical Research, Kansas City, Missouri, United States of America; 2 Department of Biochemistry and Molecular Biology, University of Kansas Medical Center, Kansas City, Kansas, United States of America; University of Pennsylvania, United States of America

## Abstract

Cohesin is a protein complex known for its essential role in chromosome segregation. However, cohesin and associated factors have additional functions in transcription, DNA damage repair, and chromosome condensation. The human cohesinopathy diseases are thought to stem not from defects in chromosome segregation but from gene expression. The role of cohesin in gene expression is not well understood. We used budding yeast strains bearing mutations analogous to the human cohesinopathy disease alleles under control of their native promoter to study gene expression. These mutations do not significantly affect chromosome segregation. Transcriptional profiling reveals that many targets of the transcriptional activator Gcn4 are induced in the *eco1-W216G* mutant background. The upregulation of Gcn4 was observed in many cohesin mutants, and this observation suggested protein translation was reduced. We demonstrate that the cohesinopathy mutations *eco1-W216G* and *smc1-Q843*Δ are associated with defects in ribosome biogenesis and a reduction in the actively translating fraction of ribosomes, eiF2α-phosphorylation, and ^35^S-methionine incorporation, all of which indicate a deficit in protein translation. Metabolic labeling shows that the *eco1-W216G* and *smc1-Q843*Δ mutants produce less ribosomal RNA, which is expected to constrain ribosome biogenesis. Further analysis shows that the production of rRNA from an individual repeat is reduced while copy number remains unchanged. Similar defects in rRNA production and protein translation are observed in a human Roberts syndrome cell line. In addition, cohesion is defective specifically at the rDNA locus in the *eco1-W216G* mutant, as has been previously reported for Roberts syndrome. Collectively, our data suggest that cohesin proteins normally facilitate production of ribosomal RNA and protein translation, and this is one way they can influence gene expression. Reduced translational capacity could contribute to the human cohesinopathies.

## Author Summary

Cohesin is a protein complex known for its essential role in chromosome segregation. However, cohesin and associated factors have additional functions in transcription, DNA damage repair, and chromosome condensation. Two human diseases, Cornelia de Lange syndrome and Roberts syndrome, are caused by mutations in cohesin. These “cohesinopathies” are thought to be caused by gene misregulation, although the role of cohesin in transcription has been enigmatic. Here we show that mutations in cohesin are associated with reduced production of the structural RNAs that are components of the ribosome in the budding yeast *Saccharomyces cerevisiae*. This causes defects in protein translation, which can explain a large fraction of the gene misregulation observed. We further show similar physiology in a human Roberts syndrome cell line. We postulate that reduced translational capacity contributes to the cohesinopathies.

## Introduction

Cohesin is a protein complex that binds to chromosomes from the time of their replication until their segregation. Cohesin creates cohesion between two sister chromatids in order to ensure their correct segregation upon division at the metaphase to anaphase transition. In addition to its essential role in chromosome segregation, the cohesin complex and its accessory factors have been shown to play roles in chromosome condensation, DNA damage repair and gene regulation. The cohesin complex is composed of four subunits: Smc1, Smc3, Mcd1/Scc1/Rad21, and Scc3/Irr1. The complex is loaded onto chromosomes by the Scc2-Scc4 complex [1], [2], [3]. In order to establish cohesion between sisters, Eco1 acetylates the Smc3 subunit of the complex [4], [5], [6]. Pds5 is required for maintenance of cohesion in G2/M [7], [8]. Cohesion is dissolved at the metaphase to anaphase transition when sisters are separated to opposite poles for inclusion in new daughter cells.

Heterozygous mutations in Smc1, Smc3 and Scc2/Nipped-B/NIPBL have been associated with the human disease Cornelia de Lange syndrome (CdLS) [9], [10], [11], [12]. Homozygous mutation of ESCO2 (yeast *ECO1*) is associated with the human disease Roberts syndrome [13]. The human diseases, referred to as the cohesinopathies, are perplexing since the developmental defects suggest that the primary dysfunction is transcription, rather than chromosome segregation [14]. Metaphase chromosomes from Roberts syndrome patients show “heterochromatic repulsion,” which refers to regions of “puffing” at heterochromatic regions around the centromeres and nucleolar organizers (rDNA) [15].

In order to better understand the molecular underpinning of the human diseases, and to further explore the cohesin network, we constructed yeast strains bearing mutations analogous to those associated with human disease [16]. Our yeast strains are haploid, so they do not genocopy the disease state. However, characterization of the cellular defects associated with the mutations may still be informative. Previous characterization of these strains revealed very few defects in chromosome segregation or the location of cohesin binding, but interestingly, two mutants (*eco1-W216G* and *scc2-D730V*) had defects in nucleolar morphology, induction of the *GAL2* gene, and chromosome condensation. Three mutant strains exhibited cohesion defects at 37°C (*eco1-W216G*, *smc1-Q843*Δ, and *scc2-D730V*). The *eco1-W216G* mutation disrupts the acetyltransferase activity of the protein toward Smc3 and is lethal at 37°C [17], [18]. Scc2 has recently been shown to participate not only in cohesin loading, but also in condensin loading [19]. Despite cohesion defects at 37°C, the growth of the *scc2-D730V* and *smc1-Q843*Δ mutants appears nearly normal.

To further characterize the mutants, we carried out gene expression profiling in rich medium and at various times following amino acid starvation. The gene expression pattern of the *eco1-W216G* mutant showed changes in over 1600 genes while the *scc2-D730V* mutant had essentially a wild-type gene expression profile. Under rich medium conditions, the gene expression profile of the *eco1-W216G* mutant suggested that protein translation was inhibited. By directly testing protein synthesis and ribosome biogenesis, we confirmed that translation was reduced. Strikingly, ribosomal RNA (rRNA) transcripts were significantly reduced in *eco1-W216G* and *smc1-Q843*Δ mutants. Since ribosome assembly is regulated at the level of rRNA [20], this could affect ribosome biogenesis. Cohesion was specifically reduced at the rDNA in the *eco1-W216G* mutant, reminiscent of the heterochromatic repulsion observed in Roberts syndrome. Importantly, protein synthesis and ribosomal RNA production were reduced in a human Roberts syndrome cell line, very similar to our yeast mutants. Taken together, our results suggest that cohesin proteins may normally promote production of ribosomal RNAs.

## Results

### Hundreds of genes are differentially expressed in the *eco1-W216G* mutant

Given the hypothesis that mutations in cohesin can affect gene expression, we undertook gene expression profiling of three strains: 1) wild-type (WT), 2) *scc2-D730V*, and 3) *eco1-W216G*. We selected conditions under which we expected many transcriptional changes to maximize the likelihood of finding transcriptional differences in the mutants. Cultures growing in log phase in rich YPD medium (time 0) were transferred to medium lacking amino acids and samples were collected for analysis at 15, 35, and 55 minutes. Three independent cultures were analyzed for each strain background. mRNA was extracted, purified, labeled, and used for hybridization to Affymetrix microarrays (Yeast Genome 2.0) to examine gene expression.

**Figure 1:**
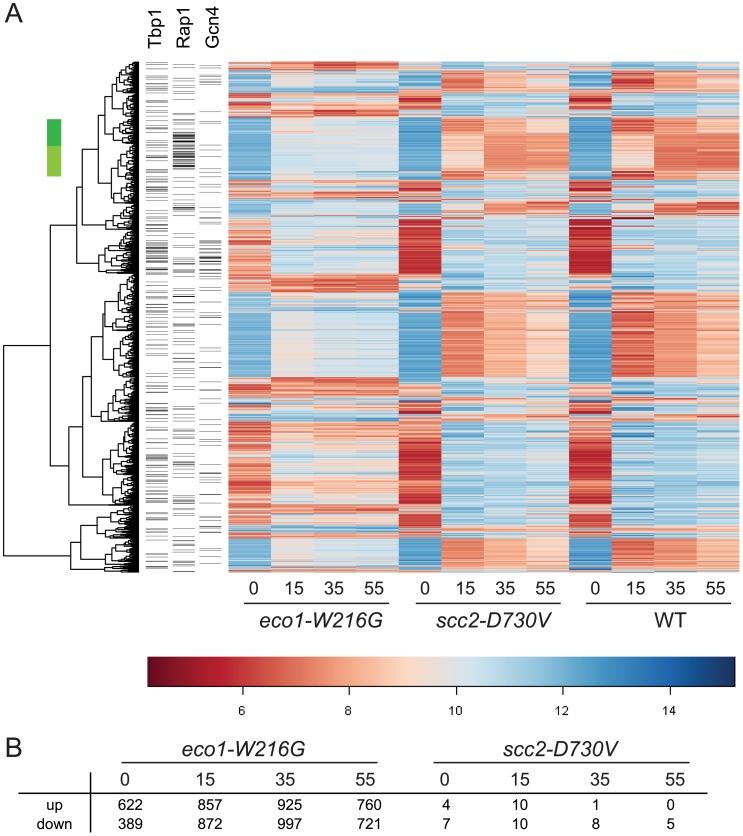
Gene expression is disrupted by the *eco1-W216G* mutation. Haploid yeast strains (WT, *scc2-D730V*, *eco1-W216G*) were grown in triplicate to mid log phase in YPD+CSM (t 0 min) and then switched to synthetic medium lacking any amino acids and timepoints were collected at 15, 35, and 55 minutes. RNA from these cultures was labeled and hybridized to affymetrix microarrays. A. Hierarchical clustering of the 1657 genes with p<0.001 for *eco1-W216G*/WT comparison. The color bar is used to indicate the log_2_ of the array intensity for each gene which corresponds to transcript level. B. Table showing the number of genes up and down regulated with an adjusted p<0.05 for each timepoint for each mutant. See Table S1 for GO analysis of differentially expressed genes. See Figure S1 for evaluation of tRNA gene mediated silencing.

To compare each mutant directly to WT, ratios were formed between each mutant and WT for each time point. Contrasts were created using limma to average replicates and determine p-values for each difference. After adjusting the p-values for multiple hypothesis testing, a set of genes was selected on the basis of adjusted p-values of less than 0.001 from any time point in either mutant/WT comparison. The result was that 1659 genes differed in expression, 1657 for *eco1-W216G* and 2 for *scc2-D730V*. Hierarchical clustering of the 1657 genes revealed the expression pattern in the *eco1-W216G* mutant was highly disrupted relative to the other two strains (Figure 1A). The number of genes up or down regulated in mutant/WT by at least 1.4 fold, with p-values of less than 0.05, for each timepoint is shown in Figure 1B. The lack of disruption in the *scc2-D730V* mutant background is notable since *scc2-D730V* and *eco1-W216G* mutant strains both have similar levels of chromosome decondensation and disrupted nucleolar morphology [16]. These results suggest that the *scc2-D730V* defects are not sufficient to cause major changes in gene expression.

We have previously reported that the clustering of tDNA adjacent to the nucleolus is disrupted in both the *scc2-D730V* and *eco1-W216G* mutant strains [16]. This clustering has been associated with the silencing of genes adjacent to tDNAs, a phenomenon referred to as tDNA gene mediated silencing [21]. We analyzed whether expression of the genes adjacent to tDNAs were misregulated in the mutants relative to WT. We found no evidence that genes adjacent to tDNAs were differentially regulated in the mutants (Figure S1), suggesting that control of gene expression via tDNA clustering is not a wide-spread phenomenon, although there still may be individual cases of gene regulation via this mechanism. Our results are consistent with previous findings showing that mutations in RNA pol III, which disrupt tDNA clustering, do not disrupt the expression of neighboring genes [22].

We performed a GO analysis on the genes differentially expressed (both up and down) with an adjusted p value less than 0.005 at time 0 (639 genes) and 15 minutes (627 genes) in the *eco1-W216G* mutant as compared to WT [23]. At time 0 we found that many of the differentially expressed genes are involved in glutamate metabolic processes, TCA cycle, cell wall organization, and acetyl-CoA metabolism (Table S1). Glutamate and glutamine are donors of amino groups for the biosynthesis of nucleotides, amino acids, and other nitrogen containing compounds. When the gene expression profile in rich medium for the *eco1-W216G* strain was compared to a variety of stress response profiles [24], it most closely matched nitrogen starvation. At the 15 minute timepoint, the enriched GO terms are almost all related to ribosome biogenesis, including biogenesis of ribosomal proteins and processing of RNAs needed for ribosome assembly (Table S1).

### The transcriptional activator Gcn4 is upregulated in cohesin mutants

**Figure 2:**
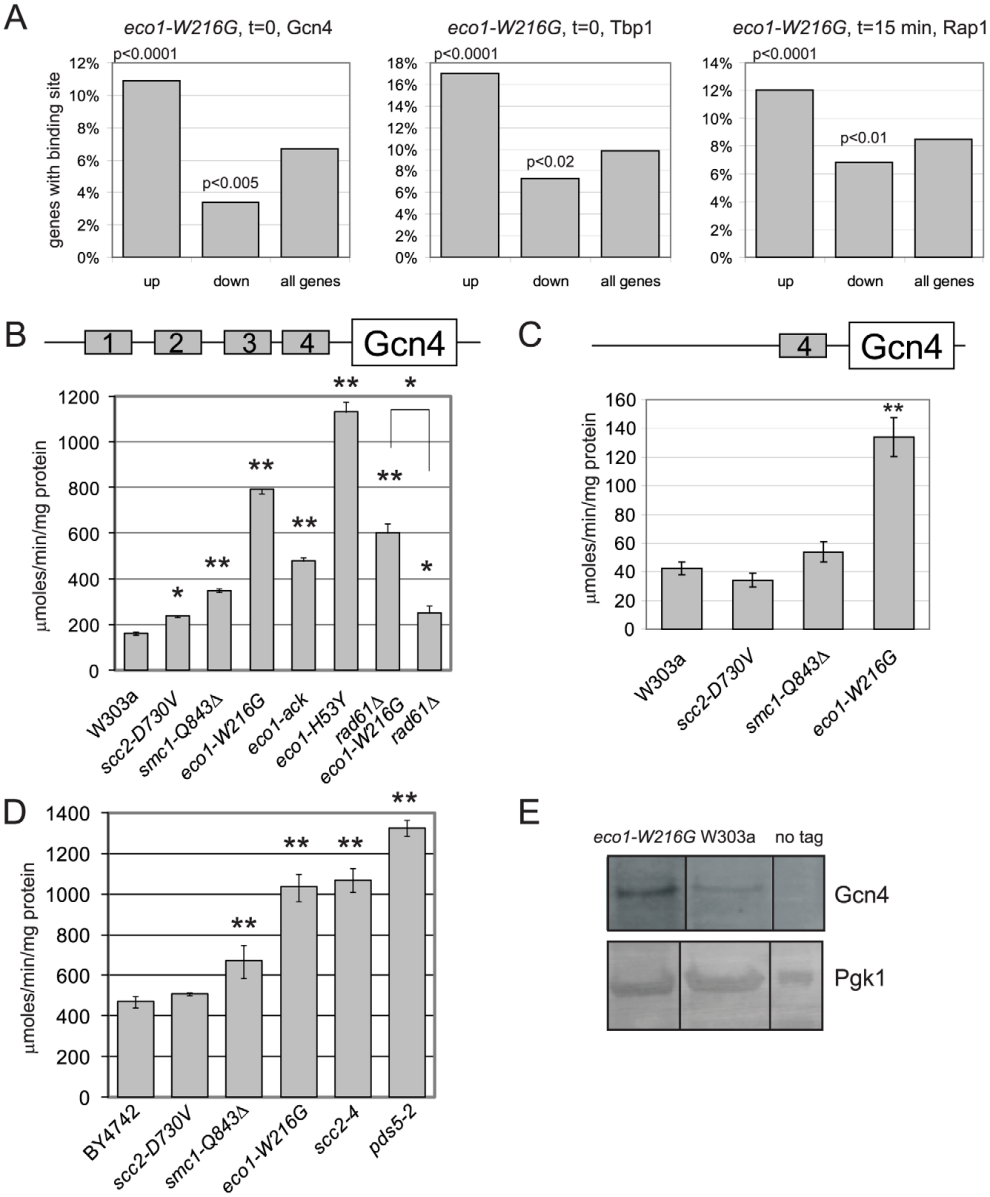
Gcn4 targets and Gcn4 are elevated in cohesin mutants. A. Histogram for transcription factor binding sites from the *eco1-W216G* strain showing the number of genes upregulated or downregulated from Figure 1B that have a Gcn4 site (time 0), a Tbp1 site (time 0), or a Rap1 site (time 15 min). The p value is calculated by a hypergeometric test using the number of up or down regulated genes with the binding site versus the number of genes in the genome with the site. B. Strains with W303 background having the indicated mutations were transformed with the p180 reporter plasmid that contains a Gcn4-lacZ transgene. β-galactosidase levels (y axis) were measured for each strain in triplicate following growth to mid log phase in YPD+CSM. The error bars represent the standard deviation of at least three independent measurements. One asterisk indicates p less than or equal to 0.002, two asterisks indicates p<0.0001 from a Student's two tailed t test. C. β-galactosidase levels were measured using the p226 reporter. This construct has only the 4^th^ uORF from the Gcn4 leader sequence, which confers minimal translational control. D. Strains with the BY4742 background with the indicated mutations were treated as in B. E. Gcn4 was tagged with the TAP epitope. Protein extracts from equal numbers of cells were used for Western blotting. Gcn4-TAP was detected with the α-PAP antibody. Pgk1 serves as a loading control. All samples were loaded on the same blot and subjected to the same exposure, but intervening lanes were removed. See Figure S2 for RT-qPCR confirmation of the misregulation of the Gcn4 targets *SNO1* and *SNZ1* in the mutants.

The gene expression data was further analyzed to determine whether the genes that were misregulated in the *eco1-W216G* mutant had any enrichment for particular transcription factor binding sites in their promoter regions. In the promoters of genes that were upregulated at the time 0 timepoint, we found a significant enrichment for Gcn4 and Tbp1/Spt15 binding sites (Figure 2A). Gcn4 is a transcriptional activator that activates the expression of many classes of genes, including stress and amino acid biosynthesis genes. Tbp1/Spt15, or TATA binding protein, is an evolutionarily conserved general transcription factor that interacts with other factors to form transcription preinitiation complexes at promoters. *SNO1* and *SNZ1* have been reported to be upregulated in pol III mutants [22] in a Gcn4-dependent manner [25]. These genes were found to be upregulated in the microarray data. The misregulation of *SNO1* and *SNZ1* was confirmed by RT-qPCR (*SNO1*, 3-fold, *SNZ1*, 9-fold, *eco1-W216G* at time 0, Figure S2). In the promoters of genes that were downregulated at time 0 there were fewer than average Gcn4 and Tbp1/Spt15 binding sites.

In the promoters of genes that were differentially expressed at the 15 minute timepoint, there was a significant enrichment for Rap1 binding sites. One group of these genes spans two clusters (Figure 1A, green bar); most of these genes are involved in ribosome biogenesis (adj p<0.001). Rap1 (Repressor Activator Protein) regulates the transcription of many ribosomal protein genes [26]. When cells are starved for amino acids, they normally repress genes involved in ribosome biogenesis [24]. While these genes were repressed once amino acid starvation was initiated in all three strain backgrounds, the genes were more weakly repressed in the *eco1-W216G* background. The reason for this is currently unclear, but may be related to the baseline ribosome defect in this strain (see below).

Gcn4 is a transcriptional activator that is normally translated only when cells encounter stress or nutritional starvation [27]. Surprisingly, the enrichment for Gcn4 binding sites in the promoters of genes upregulated in the *eco1-W216G* mutant at time 0 suggested that Gcn4 was activating the transcription of its normal target genes in the *eco1-W216G* mutant when cultures were growing in rich medium, prior to amino acid starvation. Although many Gcn4 target genes were induced in the *eco1-W216G* mutant background under rich growth conditions, the mRNA corresponding to Gcn4 was unchanged in the mutants relative to WT (see microarray data GEO GSE27235). Gcn4 contains an unusual leader sequence with four short ORFs (uORFs). One level at which Gcn4 is regulated is translation; translation of the Gcn4 mRNA occurs when ribosomes become processive due to limiting pools of GTP. For this reason, Gcn4 has been used extensively as a reporter for ribosome function [27], [28].

We used a Gcn4-lacZ reporter (p180) to determine whether β-galactosidase levels were elevated in the cohesinopathy mutants in the W303a strain background. We found a 4-fold elevation in β-galactosidase activity in the *eco1-W216G* strain as compared to a WT strain (Figure 2B). The cohesinopathy mutant *smc1-Q843*Δ also showed elevated β-galactosidase activity in this assay, while the *scc2-D730V* showed a very mild elevation. We also analyzed the β-galactosidase levels in two additional *eco1* alleles. We previously reported that *eco1-H53Y*, *eco1-W216G*, and *eco1-ack* represent an allelic series (strongest to weakest) with respect to both cohesion as measured by a 1 spot-2 spot assay, and DNA damage sensitivity [17]. Stronger cohesion defects and DNA damage sensitivity were correlated with higher levels of β-galactosidase activity. Defects in cohesion have been previously noted at 37°C for the *eco1-W216G*, *smc1-Q843*Δ, and *scc2-D730V* strains [16] and the degree of defect correlates with the β-galactosidase activity observed.

We previously showed that deletion of *RAD61*/*WPL1* rescued the growth of the *eco1-W216G* mutant at 37°C but did not rescue the X-ray sensitivity [17]. While β-galactosidase levels in the *eco1-W216G rad61* double mutant were lower than the *eco1-W216G* single mutant, they remained higher than WT, suggesting that some defect persists. Deletion of *RAD61* has been shown to partially rescue the cohesion defect of an *eco1-1* mutant [4].

We further tested whether the increase in β-galactosidase activity was dependent on the presence of uORF4 in the Gcn4 promoter using an additional reporter construct. p226 has only uORF4. The deletion of the first 3 uORFs results in very minimal translational control [29]. The elevation in β-galactosidase activity remained with uORF4 for *eco1-W216G* (Figure 2C), but the level was reduced compared to the p180 reporter, as expected if translational control is contributing to the elevation.

We also analyzed the β-galactosidase levels in the cohesinopathy mutants in the BY4742/S288C strain background, as well as *scc2-4* and *pds5-2* mutants (Figure 2D). All mutants except *scc2-D730V* showed elevated levels of β-galactosidase compared to a WT control. We conclude that mutations in many different cohesin associated genes and in two different strain backgrounds can give rise to elevated levels of β-galactosidase activity expressed from the Gcn4 promoter.

We measured Gcn4 protein levels directly by Western blotting. The *eco1-W216G* mutant strain has a higher level of Gcn4 than a wild-type strain when grown in rich medium (Figure 2E), consistent with the results from the reporter assay and the gene expression data.

### Protein translation is impaired in the cohesinopathy strains

**Figure 3:**
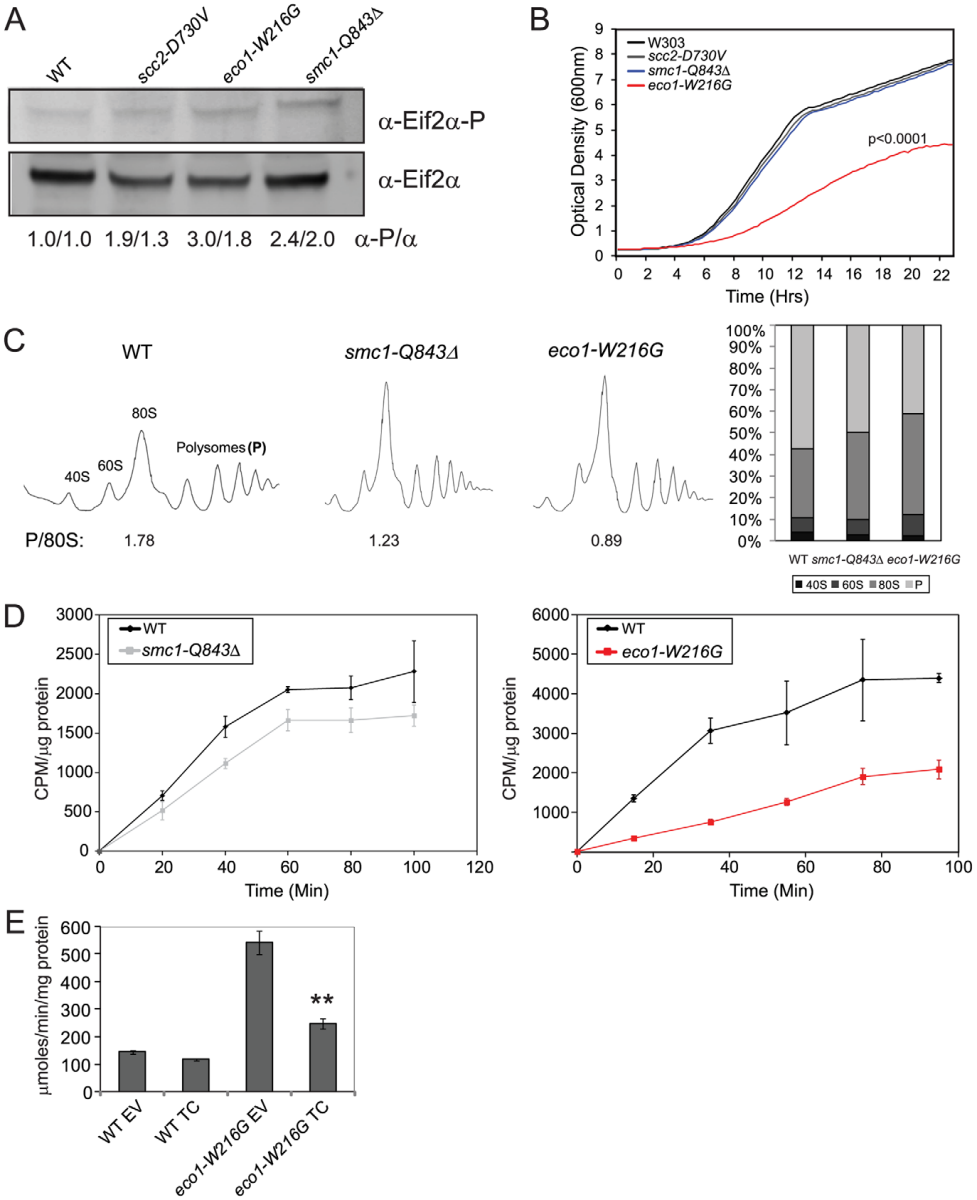
Cohesinopathy mutants display phenotypes consistent with translation defects. A. Whole cell extracts were made from a WT, *scc2-D730V*, *smc1-Q843*Δ, and *eco1-W216G* mutant strains grown in YPD+CSM at 30°C. Extracts were used for Western blotting to measure levels of eiF2α protein, and phospho-eiF2α, which is an indicator of translational inhibition. Biological replicates yielded similar results (the first number corresponds to the blot shown). B. Growth profiles are shown for WT, *scc2-D730V*, *smc1-Q843*Δ, and *eco1-W216G* mutant strains. Profiles were collected at 15 minute intervals in triplicate for each strain in YPD+CSM at 30°C; a single curve is shown. We derived the maximum slope of the curves in log phase and tested whether the slopes were significantly different for replicates of the same genotype or for WT versus mutant (for more information see Materials and Methods). None of the curves derived from a single genotype showed statistical significance between replicates. The p value for the comparison to WT is indicated where significant. C. Polysome profiles of WT, *smc1-Q843*Δ, and *eco1-W216G* mutant strains were collected from cells grown in YPD+CSM at 30°C. The ratio of polysomes to 80S (P/80S) is shown. Profiling was conducted at least twice with similar results. Quantification was carried out using Mathematica and Image J software with similar results. Results from Image J analysis are shown. D. Strains growing in log phase in SD-met+^35^S-methionine at 30°C (see Figure 5E for growth profile) were used to measure protein synthesis. We verified that the cohesin mutants are not methionine auxotrophs. E. WT and *eco1-W216G* mutant strains with the Gcn4-lacZ transgene integrated at the *TRP1* locus were transformed with either empty vector (EV) or a plasmid constitutively overexpressing the ternary complex (TC) by virtue of its high copy. Strains were grown and assayed as described in Figure 2. The difference between *eco1-W216G*+EV and *eco1-W216G*+TC was significant at p<0.0001. See Figure S3 for verification that the *smc1-Q843*Δ and *eco1-W216G* strains used throughout the manuscript are not aneuploid.

Given that high levels of Gcn4 can indicate a defect in protein translation, we tested whether protein translation was impaired in the cohesinopathy mutants. An evolutionarily conserved indicator of translational inhibition is the phosphorylation of elongation initiation factor 2α (eiF2α) [27], [30]. Phosphorylation of eiF2α inhibits the exchange of GDP for GTP in the ternary complex, blocking translation. We used Western blotting to measure the levels of total eif2α and the phosphorylated fraction. We found a 3-fold, 2.4-fold, and 1.9 fold increase in phosphorylated Eif2α in the *eco1-W216G*, *smc1-Q843*Δ, and *scc2-D730V* lysates, respectively (Figure 3A).

Since defects in translation could slow growth, we monitored growth in our cohesinopathy strains in rich medium (YPD+CSM) at 30°C. The *eco1-W216G* mutation confers a strong growth defect in the W303a background (p<0.0001). However, the growth of the *scc2-D730V* and *smc1-Q843*Δ mutant strains was not significantly different from WT (Figure 3B). Since mutations in cohesin or its regulators could cause chromosomal instability, we verified that our strains (Table S2) are not aneuploid (Figure S3).

Since growth can be affected by many different factors, we decided to analyze protein translation using more direct measures. To analyze ribosomes directly, we used sucrose gradients in combination with fractionation (Figure 3C). The ratio of polyribosomes to 80S indicates the active translating fraction. The 80S peak will consist of ribosomes without an associated mRNA or “vacant” ribosomes as well as some with an mRNA (monosomes). In theory, the polysome to 80S ratio becomes smaller with initiation defects, while it becomes larger with elongation defects [31]. The ratio of polysomes to 80S in WT, *smc1-Q843*Δ, and *eco1-W216G*, respectively, was 1.78, 1.23, and 0.89, consistent with a translation initiation defect in the mutants. The decrease in actively translating ribosomes could indicate a defect in protein synthesis.

In order to further measure protein translation, we used ^35^S-methionine incorporation to quantify protein synthesis. We found ∼50% reduction in incorporation in the *eco1-W216G* mutant and a ∼20% reduction in the *smc1-Q843*Δ mutant relative to the WT strain (Figure 3D). Collectively these results are consistent with the idea that the *smc1-Q843*Δ and *eco1-W216G* mutants support lower levels of protein synthesis.

The ribosome profiles suggested that initiation was limiting in the mutants. To test whether initiation was defective in the *eco1-W216G* strain, we transformed it with a plasmid carrying the ternary complex (eif2α, β, and γ, and tRNA-fMet) [32]. Overexpression of the ternary complex could reduce β-galactosidase levels expressed from the Gcn4 promoter if the high levels were due to poor translation initiation. We found that the plasmid reduced β-galactosidase levels in the *eco1-W216G* strain background (Figure 3E), consistent with a defect in the initiation of translation.

### Ribosome biogenesis is impaired in the cohesinopathy strains

**Figure 4:**
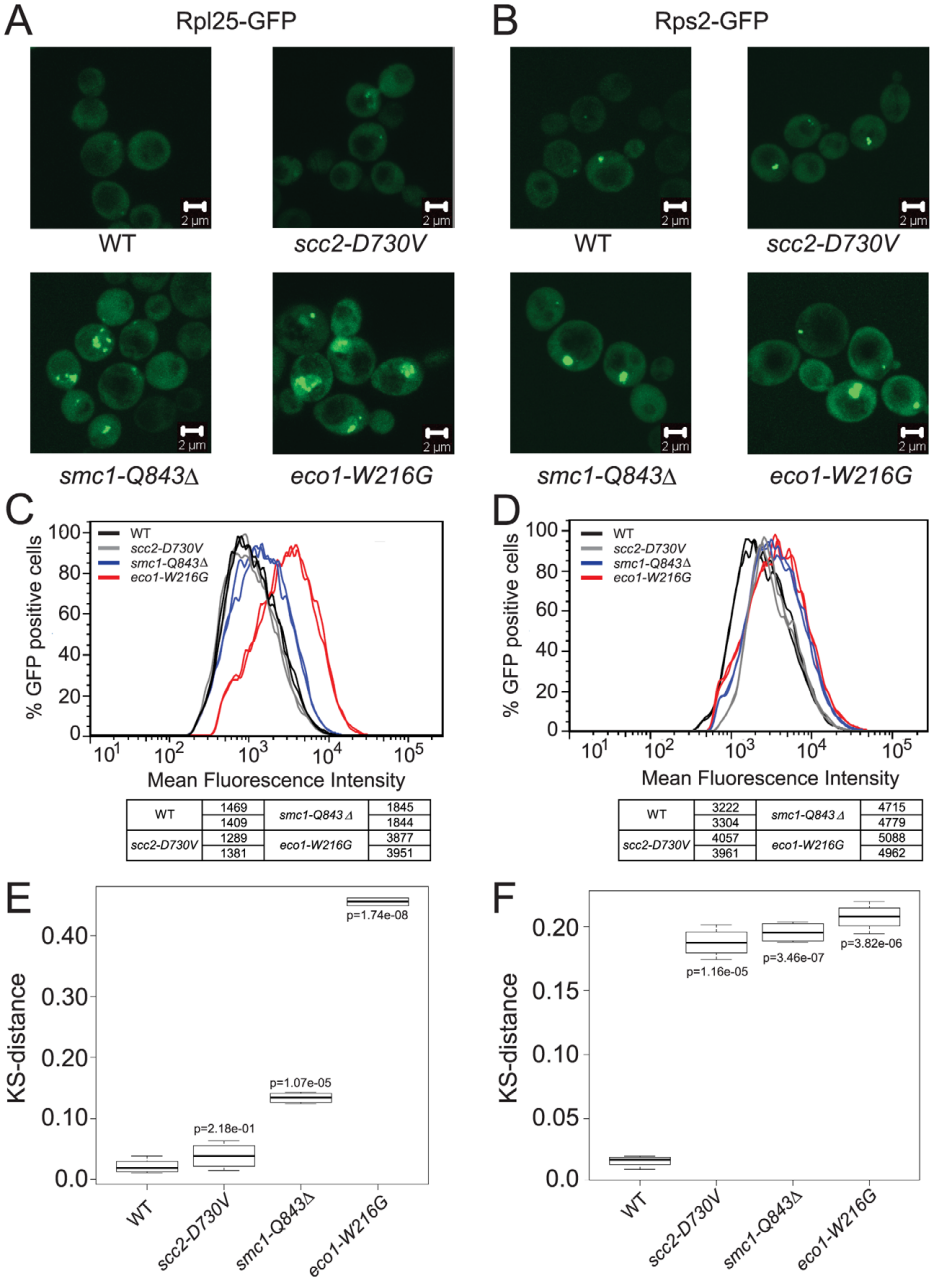
Cohesinopathy mutants show defects in ribosome biogenesis. The indicated strains in the W303a background were transformed with a plasmid carrying either a reporter for the 60S subunit, Rpl25-GFP (A) or a reporter for the 40S subunit, Rps2-GFP (B). Images of live cells were collected using confocal microscopy (LSM 510 Axiovert; Carl Zeiss, Inc) with a 100× Plan Apochromat 1.46 NA oil objective, using AIM software. In order to quantify the fluorescence intensity, approximately 10,000 cells of each genotype were subjected to FACScan analysis and the peak GFP fluorescence was measured. For each genotype at least two independent samples were measured. Cultures were grown at 30°C in SD-leu supplemented with adenine and collected in log phase. The distribution of fluorescence is shown (C, D). A KS test was applied to the distributions (see Materials and Methods) and a t test was used to determine if the distance from WT (shown as a box plot) was statistically significant (E, F).

In order to further analyze the production of ribosomes in the *scc2-D730V*, *smc1-Q843*Δ and *eco1-W216G* mutants, we transformed them with plasmids that contain GFP reporters for the assembly of the 40S (Rps2-GFP) and 60S (Rpl25-GFP) components of the ribosome. In WT cells these proteins are mainly found evenly distributed in the cytoplasm. However, if there is an assembly and/or export defect, this is visualized as an accumulation of the GFP protein in the nucleus or nucleolus [33], [34]. We collected images of our mutants transformed with these reporters and we observed the accumulation of both reporter proteins in the *smc1-Q843*Δ and *eco1-W216G* mutants (Figure 4A and 4B). To further quantify this effect we developed a cytometric approach that allowed us to monitor at least 10,000 cells per sample. When the peak GFP fluorescence was measured, the *smc1-Q843*Δ and *eco1-W216G* mutants had higher mean fluorescence for both the 40S and 60S reporters, while the *scc2-D730V* mutant showed a mild phenotype for the 40S reporter but no increase in fluorescence for the 60S reporter (Figure 4C and 4D). To further analyze the data we generated the cumulative distribution function for each sample (not shown), and then we calculated the distance between biological replicates and between mutant and WT using a KS test (see Materials and methods). These distances are depicted as a box plot with an associated p value to indicate whether the distance from WT is statistically significant (Figure 4E and 4F). In summary, both the 40S and 60S subunits of the ribosome exhibit assembly/export defects in both the *smc1-Q843*Δ and *eco1-W216G* mutants, with a more severe defect observed in the *eco1-W216G* mutant.

### Ribosomal RNA production is reduced in cohesin mutants

**Figure 5:**
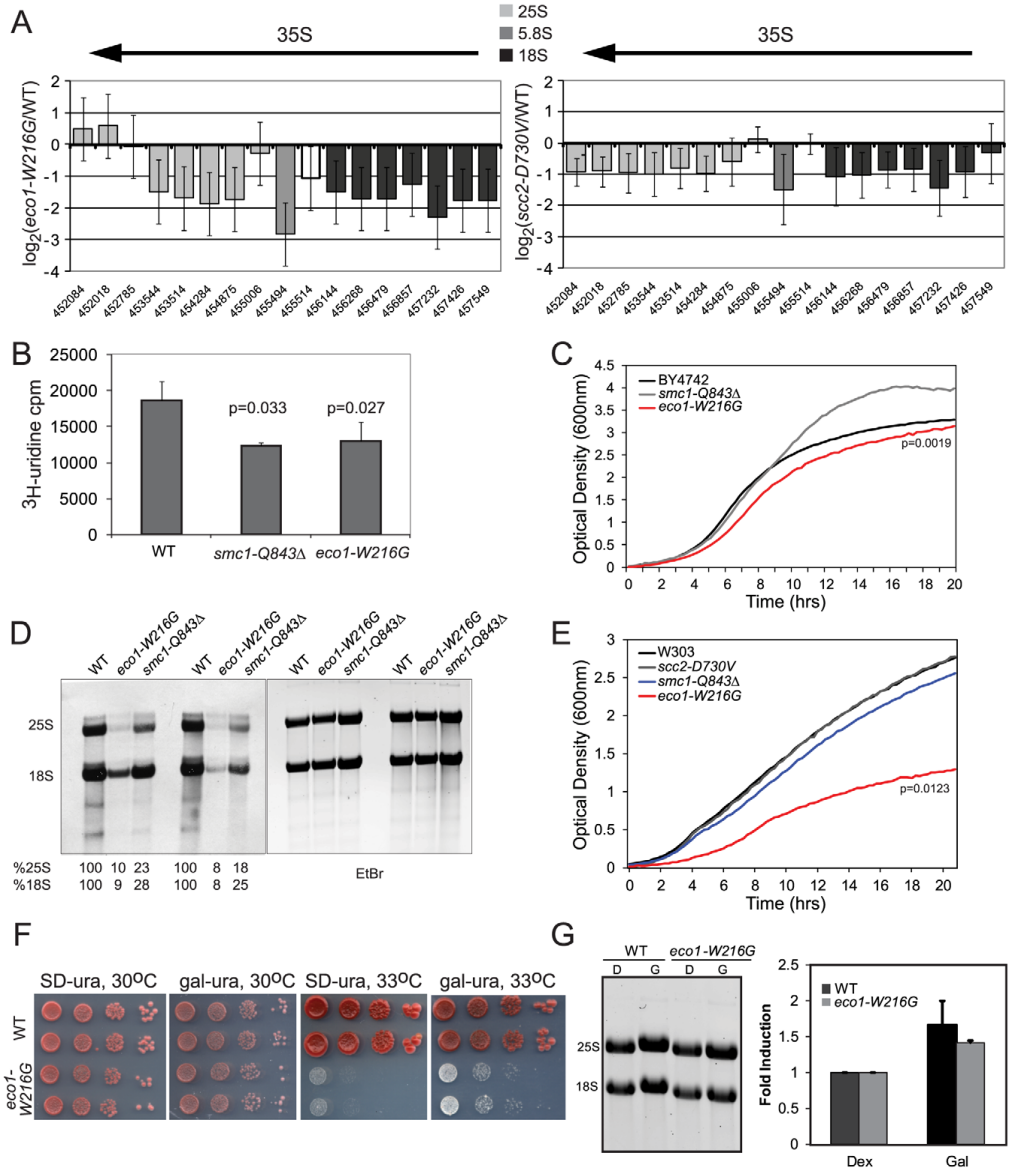
Cohesin mutations compromise production of ribosomal RNA. A. The ratios for microarray probes corresponding to the 25S, 5.8S and 18S transcripts of the rDNA locus are shown for *eco1-W216G*/WT and *scc2-D730V*/WT at time 0 from Figure 1. The x axis corresponds to SGD coordinates, ordered by the beginning of the probe with the midpoint of the probe given. The arrow indicates the direction of transcription. The error bars show the standard error. B. Strains were in log phase in SD-ura at 30°C when an aliquot was removed and ^3^H-uridine was added for 5 min to equal numbers of cells for each strain background. Incorporation was measured by scintillation counting after extensive washing of the cells. Three independent cultures were labeled to derive the standard deviation. Significance was calculated using an unpaired t test. C. A growth curve is shown for the strains in SD-ura medium at 30°C. A similar experiment was performed in the W303a background and is included in Figure S4. D. Strains were grown in SD-met at 30°C and RNA was extracted from equal numbers of cells following a 5 minute pulse with ^3^H-methylmethionine and a chase with cold methionine. Equal amounts of RNA were run on a formaldehyde gel and photographed following staining with ethidium bromide (EtBr). Then the RNA was transferred to a membrane for exposure. Following exposure, the bands were excised and radioactivity was determined by scintillation counting. Percent incorporation is given as a fraction of WT. Independent biological replicates are shown. E. A growth curve is shown for the strains in SD-met medium at 30°C. F. Growth of the *eco1-W216G* mutant at 33°C is partially rescued by expression of the rDNA from a Pol II (gal) promoter. G. Total RNA was isolated from the strains shown following growth in raffinose followed by a 5.5. hour incubation with either glucose or galactose. The total amount of 28S+18S was quantified in glucose and galactose.

We noticed from the microarray data that RNA polymerase I dependent ribosomal RNA (35S transcript) was downregulated approximately 4-fold in the *eco1-W216G* mutant (median p value 0.01, median adjusted p = 0.07) and 2-fold in the *scc2-D730V* mutant (median p value 0.12, median adjusted p = 0.40) in rich medium (Figure 5A). We note that transcripts corresponding to RNA polymerase I subunits appear to be unaffected in the transcription profile of the *eco1-W216G* mutant, suggesting downregulation of RNA Polymerase I is not causing the reduction in the 35S transcript. Notably, ribosomal RNA has been shown to be a limiting factor for ribosome assembly [20]. Since ribosomal protein genes showed no significant differences in transcription between the *eco1-W216G* mutant and WT in rich medium (Figure 1), we speculated that the ribosome defect was not due to a lack of proteins needed to make ribosomes, but possibly due to the low levels of rRNA.

Because rRNA constitutes ∼60% of the RNA being made by actively growing cells, ^3^H-uridine incorporation is commonly used to measure total rRNA synthesis. To further test the new production of rRNA, we pulsed with ^3^H-uridine and measured incorporation into RNA. In the *eco1-W216G* and *smc1-Q843*Δ mutants, there is less incorporation in 5 minutes in an equal number of cells (Figure 5B), indicating that these mutants produce less rRNA in this time frame. These experiments were carried out in the BY4742 background and the growth in SD-ura at 30°C was measured (Figure 5C). In log phase, which is when the labeling is performed, only the *eco1-W216G* mutant showed slower growth. We carried out a similar labeling experiment with the *eco1-W216G* mutant in the W303a background and obtained similar levels of incorporation (Figure S4A). In this background, growth is much more severely affected (Figure S4B). Thus, while the *eco1-W216G* mutation confers different growth defects in different strain backgrounds, the effect on total rRNA production appears to be similar, suggesting growth may not perfectly correlate with rRNA production.

RNA polymerase I produces the 35S transcript that is then processed into the 25S, 18S, and 5.8S transcripts and further modified by methylation and pseudouridylation. To measure the production of methylated rRNA, we used incorporation of ^3^H-methyl-methionine. Total RNA was isolated from equal numbers of cells following a 5 minute pulse labeling and a chase with cold methionine. Equal amounts of RNA were electrophoresed on a formaldehyde agarose gel and visualized with ethidium bromide (Figure 5D). Following exposure to film, the bands were excised and radioactivity was measured. We found that the *eco1-W216G* mutant produced 8–10% of WT levels of the methylated 25S and 18S transcripts and the *smc1-Q843*Δ mutant produced 18–28% of WT levels (Figure 5D). The growth curves for the mutants in SD-met at 30°C are shown (Figure 5E). Thus, while new production of total rRNA appears to be reduced approximately 2-fold in both mutants, the methylated form of the 25S and 18S transcripts is produced at a 10-fold lower level in the *eco1-W216G* mutant as compared to a 4-fold lower level in the *smc1-Q843*Δ mutant. The difference in production of total rRNA versus the processed and modified forms suggests that both initial production and subsequent processing are defective in the mutants, with a more severe defect in the *eco1-W216G* mutant. The fact that both 25S (60S rRNA component) and 18S (40S rRNA component) transcripts are affected in both mutants is consistent with the result that both 40S and 60S biogenesis are affected in both mutants.

A W303a strain bearing the *eco1-W216G* mutation does not grow at 33°C and cohesion is compromised at 37°C. Cohesion defects have been correlated with growth defects, and so it might be assumed that errors in chromosome segregation cause the lethality associated with mutations in cohesin. However, the *scc2-D730V* and *smc1-Q843*Δ strains have cohesion defects at 37°C, but can grow [16] (Figure S4C), suggesting precocious sister separation does not necessarily cause lethality. We tested whether transcription by RNA polymerase II of the 35S transcript from a galactose-inducible promoter would rescue growth of the *eco1-W216G* strain at 33°C. This plasmid allowed a partial rescue of the growth defect, suggesting some portion of the defect may be due to limiting levels of rRNA (Figure 5F). We further tested how much the rRNA levels increase in galactose medium and we found that the increase was a modest 40–50% (Figure 5G). However, this increase is similar in degree to the decrease in labeling observed with ^3^H-uridine, suggesting this increase should be sufficient to make up the difference. To explain the partial rescue we point out that 1) the morphology of the nucleolus is disrupted in the mutant, so even with more rRNA ribosome biogenesis may still be impaired, 2) the endogenous rDNA locus may still have defects associated with it, for instance, if there is difficulty with its replication, this defect will not be corrected by providing more rRNA and 3) at the elevated temperature there may be so little Eco1 function that other chromosomal processes such as chromosome segregation have become severely affected. A high copy plasmid with the 35S transcript produced from the normal promoter provides no rescue (data not shown). Overall our results suggest that some mutations in cohesin are associated with defects in 25S and 18S production.

### The *eco1-W216G* and *smc1-Q843*Δ mutations are associated with fewer transcripts from a single repeat

Many different cohesin mutations confer elevation in β-galactosidase levels from the Gcn4 leader sequence, suggesting the elevation is related to defects in chromosome cohesion. However, mutations in the cohesin network have been shown to affect both chromosome condensation [35] and DNA damage repair [36]. Both the *eco1-W216G* and *scc2-D730V* mutations confer defects in chromosome condensation and nucleolar morphology, but importantly, the *smc1-Q843*Δ strain does not share these defects [16]. This suggests that aberrant chromosome condensation and nucleolar morphology are not the primary cause of the reduction in rDNA transcription.

**Figure 6:**
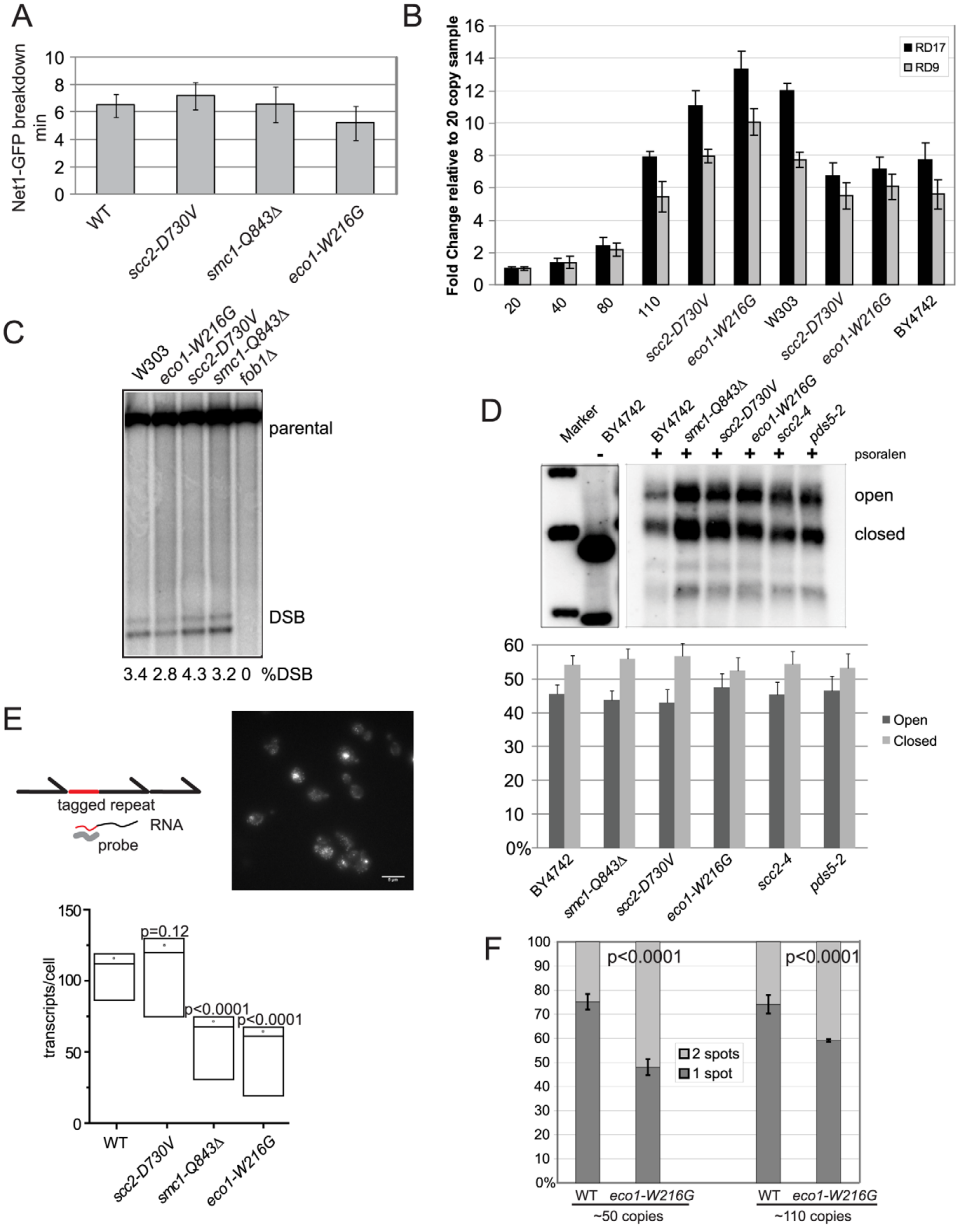
Cohesin mutations do not affect rDNA segregation, copy number, recombination, or the transcriptionally active fraction. A. The length of time for segregation of the rDNA was measured in 10 cells using live cell imaging of Net1-GFP, a nucleolar marker. The average for each strain is shown in minutes and the error bars represent the standard deviation. B. Copy number at the rDNA was measured using quantitative PCR and genomic DNA from strains having the indicated genotypes. Two different primer pairs were used (RD17 and RD9) [69]. The mean is shown and error bars indicate the standard deviation of triplicate reactions. C. Double strand breaks at the rDNA locus were monitored by Southern blot. Quantification is shown below each lane. This experiment was repeated twice with similar results. D. Psoralen crosslinking was performed followed by Southern blotting to determine the fraction of open (O) and closed (C) rDNA repeats. Quantification is shown. There were no significant difference between the mutants and WT strain. E. We used a strain in which a unique sequence has been inserted into one rDNA repeat in order to monitor its transcription by FISH [44]. A standard curve allowed us to infer the number of RNA transcripts per cell (Figure S5). A representative image from the wild-type strain is shown; the scale bar is 5 microns. For more details please see Materials and Methods. For each strain, at least three independent cultures were monitored using the protocol previously described [44] and at least 300 cells per culture were quantified. In the plot shown the dot is the average, the two lines around it are the standard error, and the lowest line is the median. The p value was derived from a two tailed Student's t test. See Figure S5 for the standard curve and expanded presentation of the FISH data. F. Cohesion was measured using strains with lacO repeats integrated adjacent to the rDNA cluster. Cultures were arrested with nocodazole. At least three biological replicates were performed, with at least 100 cells counted from each culture, and the standard deviation is shown. P values are derived from Fisher's test.

However, since condensation can affect segregation of the rDNA we decided to further examine whether the cohesinopathy mutations disrupted rDNA segregation. At the metaphase to anaphase transition, chromosomes segregate, followed by segregation of the rDNA. The segregation of the rDNA is dependent on condensin and decatenation [37]. Since the rDNA is silenced during anaphase [38], a longer anaphase could potentially account for a reduction in transcription. To measure rDNA segregation, we used yeast strains tagged with Net1-GFP (rDNA marker) and Spc42-mCherry (spindle pole body marker). The duration of rDNA separation can be calculated by the timing of the start of spindle elongation (sudden increase in the distance between the two SPBs) to fully separated Net1-GFP. In wild-type cells, rDNA separation takes an average of 6.5 minutes. We found no significant difference in the kinetics of rDNA segregation in any of the mutants (Figure 6A). Thus, delayed rDNA segregation during anaphase cannot account for the slow growth or the transcriptional defects at the rDNA.

The number of rDNA repeats can expand and contract, controlled by recombination. We considered the possibility that contraction of the rDNA was limiting transcription. We monitored the copy number of the rDNA using qPCR. To demonstrate that our assay can detect differences in copy number, we used strains containing 20, 40, 80, and 110 copies of rDNA, as estimated by pulsed field gel electrophoresis [39]. We found that the number of rDNA repeats was not significantly different from WT in the *scc2-D730V* and *eco1-W216G* mutants in either the BY4742 or W303 backgrounds. Copy number was also examined in a *smc1-Q843*Δ strain and found to be normal (data not shown). This result suggests reduced copy number cannot account for reduced transcription (Figure 6B).

The rDNA is especially susceptible to genotoxic stress. It is estimated that the rDNA incurs several DSBs per S phase which result in an average of 3.6 Holliday junctions [40]. Cohesin is known to bind to the rDNA [41], [42] and the *eco1-W216G* mutation decreases cohesin binding at the rDNA as measured by ChIP approximately 2-fold [16]. Since cohesion is important for the resolution of DNA damage, we hypothesized that the decrease in transcription at the rDNA in some cohesin mutants might be related to an inability to efficiently resolve recombination intermediates due to defective damage induced cohesion. We used Southern blot analysis to measure whether DSBs accumulate at the rDNA. The level of DSBs in cohesin mutants and a WT strain was similar, indicating unresolved DSBs do not accumulate at the rDNA in the cohesin mutant strains (Figure 6C). Thus, failure to repair the locus cannot account for the transcriptional defect.

A normal yeast cell contains 100–150 copies of the 9.1 kb rDNA repeat, about half of which are actively transcribed and half are inactive. The cell can regulate the number of active repeats and the rate of transcription since in a 20 or 40 copy strain, all the repeats are active and the rRNA is present at normal levels [39]. rDNA repeats can be differentiated by their different chromatin structures and accessibility to cross-linking by psoralen followed by Southern blot [43]. Inactive or closed gene copies contain nucleosomes and are therefore less accessible to psoralen, and migrate faster on a gel following crosslinking whereas active or open gene copies are devoid of nucleosomes and are more accessible to psoralen, and migrate slower following crosslinking [43]. To verify the method, we used a strain with 40 copies and found few closed repeats, as previously reported (data not shown) [39]. We examined whether the mutations in cohesin were affecting the fraction of open repeats. We found no reproducible change in open repeats in the cohesin mutants relative to a WT control strain, at least in asynchronous culture (Figure 6D). Thus, a steady state increase in closed repeats does not appear to account for the decrease in transcription.

Given that the copy number and fraction of open rDNA repeats do not seem to be affected in the cohesin mutants, we sought to further understand the reduction in rRNA we observed by microarray and metabolic labeling. We used a FISH assay in which a unique sequence is inserted into the 5′ end of one 35S gene (Figure 6E) [44]. The transcription of this sequence can be monitored with a fluorescent probe in individual cells to indicate the dynamics of transcription in the population. We integrated three different cohesinopathy mutations into this strain and monitored transcription. We found that transcripts made from this single repeat were present at significantly lower levels in the *smc1-Q843*Δ and *eco1-W216G* strains, but not in the *scc2-D730V* strain. Thus, when a single repeat is monitored, less rRNA is made from this repeat.

Lower production of rRNAs could potentially be explained by 1) reduced copy number, 2) fewer transcriptionally active repeats, or 3) reduced RNA production from active repeats. Collectively our data suggests that mutations in *ECO1* and *SMC1* can be associated with production of fewer transcripts from the open fraction of rDNA repeats. Interestingly, the *smc1-Q843*Δ and *eco1-W216G* mutations were associated with a ∼2-fold reduction in rRNA using either the ^3^H-uridine labeling method to detect total rRNA or FISH to detect a single repeat. However, the *eco1-W216G* mutant showed a 10-fold reduction in the production of the methylated rRNA while the *smc1-Q843*Δ mutant showed a 4-fold reduction. This difference correlates well with the degree of defect in protein synthesis and ribosome biogenesis. We speculate that due to the disruption in nucleolar morphology in the *eco1-W216G* mutant [16] that processing and modification of the 35S transcript as well as ribosome assembly and export might be more severely affected than in the *smc1-Q843*Δ mutant, with the outcome that translation and growth are more affected.

We have previously measured cohesion at three loci in the *eco1-W216G* mutant. We observed a 15% reduction at an arm locus, a 9% reduction at a telomere locus, and an 8% reduction at a pericentric locus relative to a WT strain, and no defect in chromosome transmission [16], [17]. However, when we measured cohesion using strains with lacO repeats integrated adjacent the rDNA [39], cohesion is reduced ∼25% in the *eco1-W216G* background in the 50 copy strain (Figure 6F). Thus, Eco1 acetyltransferase activity is differentially required for genomic and ribosomal DNA cohesion. The mechanism for this is currently unclear and will require more investigation. However, we speculate that the decrease in cohesion at the rDNA is related to the reduced transcription at this locus.

Furthermore, the specific defect in cohesion at a heterochromatic region is reminiscent of the heterochromatic repulsion observed in cells from Roberts syndrome patients [45].

### Human Roberts syndrome fibroblasts display similar physiology to yeast

**Figure 7:**
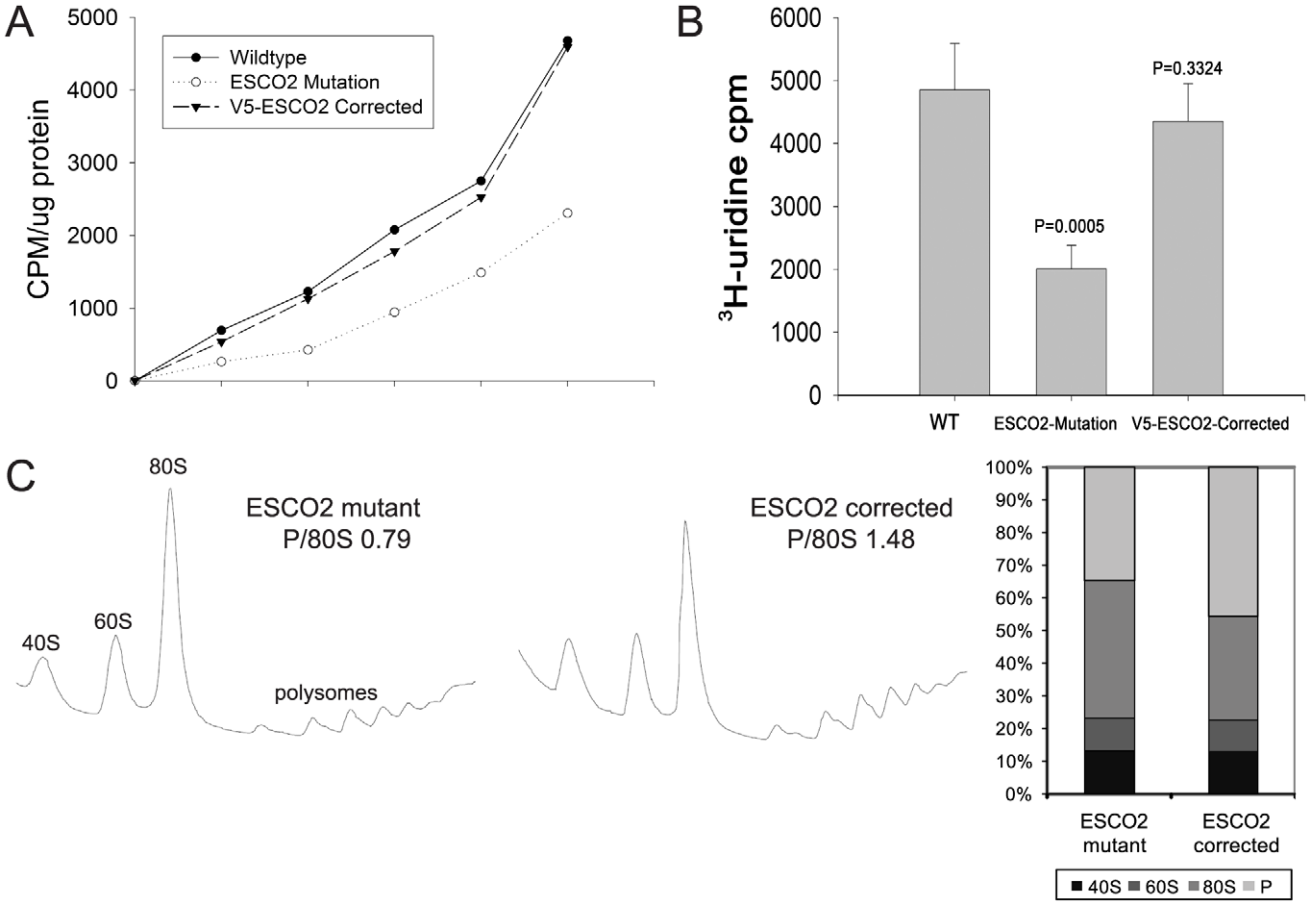
Metabolic labeling of Roberts syndrome fibroblasts suggests protein translation and ribosomal RNA production are reduced. A. Cultured WT, ESCO2-mutation and V5- ESCO2-corrected human RBS fibroblasts were grown in F10 Ham Mixture plus 10% FBS. Cells were washed in PBS twice, switched to 3 mL Met/Cys-free Dulbecco's modied Eagle's medium containing 10 µM MG-132, a proteasome inhibitor, and pulsed with 30 µCi of ^35^S-methionine for different times (0, 15, 30, 60, 120, 240 min). Cells were lysed in RIPA buffer and proteins were precipitated by the addition of hot 10% TCA. After centrifugation, the precipitate was washed twice in acetone. The precipitate was dissolved in 100 µL of 1% SDS and heated at 95°C for 10 min. An aliquot of the SDS extract was counted in Esoscint for ^35^S radioactivity in a liquid scintillation spectrometer to determine the amount of ^35^S-methionine incorporated into proteins. B. Cultured WT, ESCO2-mutation and V5- ESCO2-corrected human RBS fibroblasts were grown in F10 Ham Mixture plus 10% FBS. ^3^H-uridine (5 µCi) was incubated with 10^6^ cells from each group for two hours. Total RNA was isolated with TriZol reagent (Invitrogen, U.S.A) and the concentration of each RNA sample was measured by OD_260/280_. 1 µg of each sample was counted in a Beckman LS 6500 multipurpose scintillation counter to determine the amount of ^3^H-uridine incorporated. Four independent cultures were labeled to derive the standard deviation. Significance relative to WT was calculated using an unpaired t test. C. Ribosome profiling and quantification were carried out as described in Figure 3C.

Given that the *eco1-W216G* mutation is associated with reduced protein translation and rRNA production in budding yeast, we decided to investigate whether a human cell line bearing the same mutation displays similar physiology. We used ^35^S-methionine labeling to measure protein synthesis in 1) a Roberts syndrome fibroblast cell line, 2) a version of the cell line that has been corrected with a wild-type copy of ESCO2 [45] and 3) a normal fibroblast line. We found that the Roberts syndrome cells incorporated methionine at about 50% the level as the corrected line or a normal fibroblast line (Figure 7A), very similar to the observations in yeast (Figure 3D). Furthermore, we measured the incorporation of ^3^H-uridine as an indicator of ribosomal RNA synthesis. We found that the rate of incorporation in the Roberts syndrome cells is about 50% the level as the corrected line or a normal fibroblast line (Figure 7B), very similar to the observation in yeast (Figure 5B). Finally, we examined the polysome profile in the Roberts cells. We find that the polysome to 80S ratio is lower in the Roberts cells relative to the corrected line (Figure 7C), similar to the observation in yeast (Figure 3C). Thus, it appears that protein synthesis and ribosomal RNA production are reduced in human Roberts syndrome fibroblasts, and suggests that the findings in yeast are relevant to the human disease.

## Discussion

Several groups working in fish, flies, mouse, and humans have shown that cohesin associated mutations or reductions in cohesin associated genes result in hundreds of small alterations in gene expression, and a few cases of big changes in gene expression [46], [47], [48], [49]. These changes in gene expression are thought to cause the human cohesinopathies. However, the mechanism by which mutations in cohesin-associated genes alter gene expression has been elusive. Cohesin together with CTCF [50] or mediator [51] may facilitate gene looping and communication between promoters and enhancers [52] to influence transcription by RNA polymerase II. Cohesin may also act directly at certain loci in an activating manner [47], [49] or a repressive manner [53] to regulate transcription by RNA polymerase II . In this report, we suggest a key locus at which cohesin proteins may influence transcription is the ribosomal DNA. Misregulation at this locus can affect the transcription of hundreds of genes as translation is affected.

The elevation in Gcn4 targets suggested that this transcriptional activator was induced in the *eco1-W216G* strain, and further suggested that translation would be repressed. Analysis using a Gcn4-lacZ transgene revealed that mutations in Pds5, Scc2, Eco1, and Smc1 all showed an increase in expression, consistent with the idea that cohesion defects correlate with reduced protein translation. Also consistent with our findings, inactivation of Mcd1/Rad21 in budding yeast in G1 was shown to affect the expression of 29 genes, including genes involved in rRNA maturation and ribosome biogenesis [54]. The differential effect of the *eco1-W216G* mutation on cohesion at the rDNA is notable since the rDNA in budding yeast has many properties of heterochromatin and lack of cohesion specifically in heterochromatic regions, including the rDNA, is a hallmark of Roberts syndrome. Thus, a cohesion deficit at the rDNA is common to both our yeast model and Roberts syndrome cells. The local cohesion defect at the rDNA in the *eco1-W216G* mutant is associated with the production of fewer 35S RNA products and reduced translation. A human Roberts fibroblast line displays similar physiology to our yeast mutant in that both protein synthesis and ribosomal RNA production are impaired, suggesting yeast may provide a good model for these particular defects.

We have characterized three different cohesinopathy mutants in yeast which have overlapping sets of defects. The mutation with the strongest phenotype is *eco1-W216G*, which confers defects in nucleolar morphology, DNA damage response, growth, condensation, gene expression, ribosome biogenesis and rRNA production (this work, [16], [17]). The *smc1-Q843*Δ mutant shares the defects in ribosome biogenesis and rRNA production, albeit less severe, and without much effect on growth. If one extrapolates to multicellular organisms, one can imagine that the developmental outcomes for the RBS allele could be more severe compared to the *SMC1* CdLS allele. This proposal is consistent with observations made in zebrafish in which *ESCO2* and *RAD21* depletion were compared and *ESCO2* depletion was uniquely associated with poor cell proliferation and cell death [55]. The *scc2-D730V* allele does not have the same effect on protein synthesis as the *SMC1* and *ECO1* mutations, instead exhibiting defects in nucleolar morphology and chromosome condensation. These defects could potentially be explained by the requirement for Scc2 for condensin loading [19]. The *scc2-D730V* mutation in the W303a background does show weak elevation in β-galactosidase activity from the Gcn4 leader sequence and eif2α-phosphorylation, and a weak 40S biogenesis defect. The *scc2-D730V* mutation may affect some aspect of chromosome biology that we do not currently understand or cannot be fully evaluated in budding yeast. We note that the *scc2-4* mutation causes more severe defects in yeast (Figure 2 and data not shown). A future challenge will be to achieve a molecular understanding of how different mutations in different proteins can lead to similar disease outcomes.

In RBS both copies of *ESCO2* have lost function, but CdLS is most often caused by a single mutant copy of *SCC2*/*NIPBL*. However, *SMC1* is on the X chromosome in humans and the cases of CdLS associated with the *smc1-Q843*Δ allele have been in males with a sole mutant copy [10]. Thus, both our haploid yeast and human patients express only mutant copies of *ECO1*/*ESCO2* or *SMC1*. In contrast, the evaluation of the *scc2-D730V* allele in haploid yeast does not genocopy the human disease since there would be an additional WT copy of *SCC2*/*NIPBL* present. It may be important to model haploinsufficiency to understand how the *SCC2/NIPBL* mutations cause disease.

Since defects in protein translation affect cell growth and division, protein translation can affect size. The reports of small size in a mouse model [48] and human CdLS patients [14] are consistent with our hypothesis that mutations in cohesin can generate a deficit in ribosome function. In a report on gene expression in Drosophila cells depleted for Nipped-B or Rad21 (CdLS model), nearly all ribosomal protein and aminoacyl-tRNA synthetase transcripts are reduced [46]. In addition, expression of Myc, p53 and Mdm2 are altered by depletion of Rad21 and Nipped-B in humans [47], mouse [48], flies and zebrafish [49]. These targets are known to be regulated by ribosome biogenesis [56]. When these data are taken in context of our current report, they collectively suggest that reduced translational capacity may contribute to the developmental defects associated with the cohesinopathies.

How do cohesin proteins facilitate rRNA production? Our data suggests that transcription from a given repeat is reduced in the *eco1-W216G* strain, rather than there being fewer open repeats or reduced copy number. One mechanism by which we can imagine cohesin contributing to transcription is through gene looping, which might facilitate reloading of RNA Polymerase I from the 3′ to 5′ end of the 35S transcript. Loops at the rDNA have been reported [57] and cohesin binds flanking each repeat in a pattern that would enable looping [41]. Other possibilities include cohesin promoting some other aspect of rDNA metabolism such as replication fork speed [58] or nucleolar organization that in turn facilitates the production of the rRNA transcripts. In any case, the lower levels of ribosomal RNA as measured in both yeast and human are associated with decreased protein synthesis. In future work it will be important to further examine the mechanism by which cohesin proteins promote production of rRNA. Coupling protein synthesis capacity to chromosome metabolism might provide the cell with a useful feedback loop for regulating proliferation.

## Materials and Methods

### β-galactosidase assays

Wild type and cohesin mutant strains transformed with the plasmids p180 (*pGCN4 URA3 lacZ CEN*) having all four μORFs or p226 (with only the fourth μORF) [29] were grown at 30°C to an A_600_ of ∼0.8 under repressive conditions (overnight growth in SD-ura then shifted to YPD+CSM till desired absorbance is reached). The cells were pelleted and protein extracts were made. β-galactosidase activity was measured following standardized protocols using ONPG (o-nitrophenyl-β-D-galactopyranoside) as the substrate. We note that the level of β-galactosidase activity is very sensitive to the growth protocol used [27].

### Microarray methods

Concentration and quality of RNA were determined by spectrophotometer and Agilent bioanalyzer analysis (Agilent Technologies, Inc., Palo Alto, CA). For array analysis, labeled mRNA was prepared from 300 ng of total RNA using the MessageAmp III RNA Amplification kit (Applied Biosystems/Ambion, Austin, TX) according to the manufacturer's specifications. Array analysis was performed using Affymetrix GeneChip Yeast Genome 2.0 Arrays processed with the GeneChip Fluidics Station 450 and scanned with a GeneChip Scanner 3000 7G using standard protocols. Resulting CEL files were analyzed using RMA [59] and limma [60] in the R statistical environment. Affymetrix GeneChip data are available at GEO under accession number GSE27235.

Motif identification and analysis for Gcn4, Tbp1, and Rap1 were based on presence or absence calls for each binding site within the region of the annotated gene start site and 400 bp upstream. Presence of the Gcn4 motif was determined by a match to the sequence TGA(C/G)TC(T/A). The Tbp1 and Rap1 matches were determined using the TRANSFAC [61] matrices F$TBP_Q6 and F$Rap1_C and the MATCH program [62]. The score cut-off profiles for Tbp1 and Rap1 were minFP and minFN, respectively, using TRANSFAC version 2009.3. Sequences, microarray probe mapping, and gene annotations were from Ensembl 56. P-values for the gene set indicated were determined using the hypergeometric test of all protein-coding genes.

### Polysome analysis

100 ml of yeast culture was grown to an OD_600_ of 0.8 and treated with 100 µg/ml of cycloheximide for 10 mins on ice before centrifugation. After centrifugation the cell pellets were washed twice and resuspended in lysis buffer (10 mM Tris-HCl pH 7.5, 100 mM NaCl, 30 mM MgCl_2_, 100 ug/ml cycloheximide, 0.2 mg/ml heparin in DEPC). The cells were lysed in the cold by bead beating and the lysate (10 OD units) was loaded on top of an 11 ml 7–47% sucrose gradient in 15 mM Tris-Cl pH 7.4, 140 mM NH_4_Cl and 7.8 mM MgOAc-4H_2_O centrifuged at 36,000 rpm for 3 h. The gradients were fractionated and OD_254_ was monitored using an ISCO UV-6 monitor [63].

### Polysome quantitation

Polysome quantitation was done by both Image J and Mathematica, version 7.0. A common baseline was chosen and the area under the peaks was calculated with the Image J software. For Mathematica, TIFF images from the instrument were read using the Import function. The signal intensity was isolated from the image using the ImageSubtract function of the non-signal colors. The signal plot was scaled and shifted along the y-axis to position the baseline (x = 0) at the lowest signal level of the plot. Boundary regions were selected manually by zooming onto the image and recording the x-axis coordinate of extremal point. The total area between the selected boundaries and above the baseline was calculated using the Take and Total functions.

### Growth curves

Growth curves were collected in triplicate for each genotype at time intervals of 15 min. We used a Tecan Infinite M200 Pro machine. Due to the non-linearity between optical density (OD) and cell number at higher cell densities, the measured Tecan ODs were converted to ‘real’ ODs using the calibration function ‘real OD’ = −1.0543+12.2716×measured OD [64]. The maximum slope was determined for each curve from 12 consecutive points and the statistical significance between slopes was calculated using a t test.

### GFP measurements using cytometry

We used flow cytometry to quantify the peak GFP fluorescence in WT and mutant cells. By measuring the digitized pulse height from the B1 detector (525/50 emission), the maximum GFP intensity of each cell could be ascertained. For each sample approximately 10,000 cells were measured. WT and mutant strains that did not bear the GFP-plasmid showed no significant difference in their maximum fluorescence intensities, indicating they have similar levels of intrinsic fluorescence (autofluorescence); however, some mutant strains expressing a GFP tagged ribosomal subunit had on average a higher maximum GFP intensity than WT cells expressing the same fluorescent tag. Since the distribution of fluorescence intensity among GFP positive cells was non-Gaussian, we used the Kolmogorov-Smirnov (KS) statistic to characterize the distribution differences, which quantifies the distance between empirical cumulative distribution functions of two samples. Using this statistic, we can calculate distances between biological replicates (same genotype) and distances between samples with different genotypes. In this way we can determine whether the average KS-distance between the WT and mutant samples is significantly greater than between replicates (same genotype) using a t test.

### Western blotting

Overnight cultures of yeast cells were diluted in YPD+CSM to an OD of 0.1 and grown to an OD of approximately 0.8. Cells were then pelleted by centrifugation and washed in PBS. Cells were lysed with glass beads in buffer containing 10 mM Tris, pH 7.4, 100 mM NaCl, 1 mM EDTA, 1 mM EGTA, 1 mM NaF, 20 mM Na_4_P_2_O_7_, 2 mM Na_3_VO_4_, 0.1% SDS, 0.5% sodium deoxycholate, 1% Triton-X 100, 10% glycerol, and 1 mM PMSF protease inhibitor cocktail (Sigma). Equal amounts of protein were loaded for each sample after quantification by Bradford assay. Protein samples were electrophoresed on a 12% SDS-polyacrylamide and transferred to nitrocellulose filters. The immunoblots were probed for phospho-specific eIF2α (Ser-51) (Cell Signalling #9721). Total eIF2α was measured with rabbit polyclonal antibody (a gift from T. Dever). eIF2α was visualized by HRP-conjugated anti-rabbit IgG.

### Metabolic labeling—RNA

Methods for RNA labeling were derived from a previous report [65]. For the experiment in Figure 5B, triplicate cultures of BY4742, *eco1-W216G* and *smc1-Q843*Δ carrying pRS316 were grown in SD-Ura medium to exponential phase (OD_600_∼0.3). ^3^H-uridine (5 µCi) was mixed with 500 µL of each culture and incubated at 30°C for 5 min with aeration. Then samples were treated with 2.5 mL of 10% trichloroacetic acid (TCA) with 2.5 mg/ml of uridine. After filtration through a 25 mm glass filter, each membrane was washed with 5% TCA, dried, and counted in a Beckman LS 6500 multipurpose scintillation counter. For labeling with ^3^H-methylmethionine (Figure 5D), we grew cells in SD-met medium and pulse-labeled for 5 min with 20 µCi/mL ^3^H-methylmethionine followed by a 5 min chase with cold methionine. RNA was prepared from 1×10∧7 cells. 8 µl of each sample was run on a 1.2% formaldehyde agarose gel, transferred to a Gene Screen membrane, and detected by autoradiography. Individual RNA species (25S and 18S) were excised from the blot (together with nearby regions of the blot for assessment of background) and quantified with a scintillation counter.

### Metabolic labeling—protein

Yeast strains were grown to mid-log phase (OD_600_∼0.5) at 30°C in medium containing dextrose (YPD+CSM). Cells were harvested and washed in PBS and resuspended in a similar volume of prewarmed methionine-minus medium containing dextrose (SD-met). Aliquots were taken from this culture (0.75 ml) for the zero time point. The medium was supplemented with 27.5 µCi of ^35^S-methionine and unlabeled methionine 1 mg/ml. At 15–20 mins intervals (0.75 ml) samples were withdrawn from an actively growing culture. The amount of ^35^S-methionine incorporated into proteins was then measured by an adaptation of the method of Kang and Hershey [66]. The cells were lysed in 1.8 N NaOH containing 0.2 M β-mercaptoethanol. Proteins were precipitated by the addition of hot 10% trichloroacetic acid. After centrifugation, the precipitate was washed twice in acetone. The precipitate was dissolved in 100 µl of 1% sodium dodecyl sulfate and heated at 95°C for 10 min. An aliquot of the SDS extract was counted in Ecoscint for ^35^S radioactivity in a liquid scintillation spectrometer to determine the amount of ^35^S-methionine incorporated into proteins.

### Psoralen cross-linking

Psoralen cross-linking experiments were carried out as previously described [67] with the following modifications: 1.3% Tris-Taurine-EDTA (TTE) gels were run at 80 volts for 20 hours in 0.5× TTE, processed and transferred to Gene Screen membrane in 6× SSC. Hybridization with a 35S specific probe was carried out at 60°C and the membrane was exposed for 2 hours to a phosphorimager screen (GE/Amersham).

### qPCR for aneuploidy

Genomic DNA was isolated from strains and used as a template for qPCR. For each chromosome arm, one locus, usually near the centromere, was monitored according to the method previously described [68].

### FISH

Yeast cells were grown in CSM-URA at 30°C to an OD_600_ of 0.4. The cells were then fixed by adding formaldehyde to a final concentration of 4% (v/v) for 45 min at room temperature with shaking. After three washes with wash buffer (1.2 M sorbitol, 0.1 M potassium phosphate, pH 7.5), the cell wall was digested with 0.3 mg/ml zymolase in spheroplast buffer (1.2 M sorbitol, 0.1 M potassium phosphate, 10 mM vanadyl ribonucleoside complex, 0.06 mg/ml PMSF, 28 mM β-mercaptoethanol) at 37°C for 45 min. After digestion, the cells were washed three times with FISH wash buffer (30% formamide, 2×SCC). The cells were then hybridized in 30 µl hybridization solution containing 5 ng/µl DNA probe in 25% (v/v) formamide, 2×SCC, 1 mg/ml BSA (nuclease free), 10 mM vanadyl ribonucleoside complex, 0.5 mg/ml salmon sperm DNA and 0.1 g/ml dextran sulfate overnight at room temperature. Before imaging, cells were washed twice with FISH wash buffer for 30 min and then added to slides pre-coated with poly-L lysine.

The probe used for the FISH experiment is a synthesized DNA oligonucleotide modified from the previous publication [44]. The sequence of the oligonucleotide is 5′-CGGCRGGTAAGGGRTTCCATARAAACTCCTRAGGCCACGA-3′; the ‘R’s indicates an amino-dT replacing a regular dT where a fluorescein molecule was coupled. The probe was further purified by polyacrylamide gel purification to ensure that each amino-dT was coupled with a fluorescein molecule.

For counting RNA levels, it was first necessary to derive a calibration plot that relates intensity observed to RNA levels. This is required due to a wide range of RNA levels observed between different cells. In cells with more than ∼20 RNA, RNA spots overlap, making it impossible to distinguish individual RNAs. Extreme examples of this occur when cells have undergone recent ‘bursts’ in transcription (see Figure 6).

The general method previously developed was followed [44]. To generate a calibration plot, we acquired long-exposure z-stacks of RNA using the widefield module of a Zeiss-200 m that was also equipped with a Yokagawa CSU-10 spinning disc. For cells with few (generally less than 15) RNAs, it was possible to use the long-exposure images to count single RNAs. After counting RNAs in these cells, we switched to the confocal set-up and acquired a confocal z-stack as described below. This iteration allowed for the generation of a calibration plot that related overall intensity of the sum projection of the confocal z-stack to the number of counted RNA per cell. We obtained a linear plot, with an intercept at ∼0, demonstrating that RNA per cell is linearly proportional to total RNA, and thus total intensity per cell from the confocal data can be used to measure total RNA per cell even in cells where density is too high to distinguish single RNAs.

To acquire RNA per cell for groups of where the range is between 0 and ∼300, it was necessary to develop a system where it was possible to obtain fluorescence from cells with few (1 to 10) RNA, but yet not saturate the camera with cells possessing up to hundreds of RNA. We acquired 30 z-slices with spacing 0.3 microns. A background was subtracted for each slice, and then a sum projection was applied. Total intensity per cell was compared to the linear, extrapolated calibration plot to generate RNA per cell. A sum-projection of a non-background subtracted z-series was able to detect the location of cells where RNA levels were very low, eliminating the risk of missing low RNA-possessing cells with the background-subtracted analysis. We note that this method generated a distribution of RNA per cell that matched very closely the published result for the same strain [44].

Emission from the confocal z-slices was collected through a 500–550 nm bp filter onto a Hamamatsu C9100-13 EMCCD. A 488 nm laser line was used to excite the flourescein tagged FISH probe.

## Supporting Information

Figure S1 Genes adjacent to tDNAs are not misregulated in cohesin mutants. We examined the expression of genes adjacent to tRNAs in the microarray data set. The coordinates for all yeast tRNA genes were retrieved from Ensembl (299 genes). A script was written to use the Ensembl API and select the nearest gene to the left and to the right of each tRNA. The result was that 35 segments to the left or right of a tRNA gene did not have another gene adjacent within 2 kb, and 67 of the genes returned were another tRNA gene. The remaining gene IDs were mapped against the Affy probe IDs resulting in 418 matches. The mutant/WT expression values for this gene set for each timepoint are mapped in the box plot. In addition, a background set of mutant/WT expression values from 418 randomly chosen genes is also shown. The distribution of expression values from each mutant relative to WT for genes adjacent to a tRNA is shown (tA – tRNA Adjacent). Next to each of these distributions is a set of values from a randomly chosen gene set (RS) from the same dataset. If disruption of the tRNA suppression effect occurs in the mutants, then we would expect to observe an upward shift in the tA distribution relative to the RS set (indicating higher expression of tRNA adjacent genes in the mutant).
(PDF)

Figure S2 *SNO1* and *SNZ1* misregulation in cohesin mutants is confirmed by quantitative PCR. RT-qPCR was performed on the RNAs from each timepoint for WT and the two mutants. Gene specific primers for *SNZ1* (A) and *SNO1* (B) were used and the increase over time was calculated relative to *ACT1* and *PGK1*. Both *SNZ1* and *SNO1* are more strongly induced in the mutants. The fold change relative to the WT value is shown for the *eco1-W216G* mutant at time 0 since the scale makes the change difficult to appreciate. Reactions were performed in triplicate and the mean and the standard deviation is shown.
(PDF)

Figure S3 qPCR assay for a sequence on the left and right arm of each chromosome confirms that no aneuploidy is present in either the W303a strain or the *smc1-Q843*Δ (SG136) and *eco1-W216G* (SG156) mutants derived from this strain.
(PDF)

Figure S4 Growth and rRNA labeling. A. RNA was pulse labeled and the incorporation of ^3^H-uridine was quantified in W303a and *eco1-W216G* strains as performed in Figure 5. B. The growth of the strains used for (A) in SD-ura at 30°C is shown. C. The growth of the strains indicated is shown in YPD+CSM at 37°C. The growth curve and statistics for B and C were performed as in Figure 3.
(PDF)

Figure S5 An expanded presentation of the FISH data. A. The standard curve shown was used to determine how fluorescence intensity relates to number of RNAs. The fluorescence for each cell is measured and then binned to show the fraction of the population with each RNA number. The fluorescence for 300 cells from 3 independent cultures for each strain was measured (900 cells total per strain). The error bars indicate the standard deviation. B. WT C. *scc2-D730V* D. *eco1-W216G* E. *smc1-Q843*Δ.
(PDF)

Table S1 GO analysis of misregulated genes.
(XLSX)

Table S2 Strains.
(XLSX)
