# Supplementary figures and images for: Cohesin Proteins Promote Ribosomal RNA Production and Protein Translation in Yeast and Human Cells
Source: PLoS Genet. 2012 Jun 14;8(6):e1002749. doi: 10.1371/journal.pgen.1002749 (PMC3375231; doi:10.1371/journal.pgen.1002749)

## tRNA associated genes vs Random Set

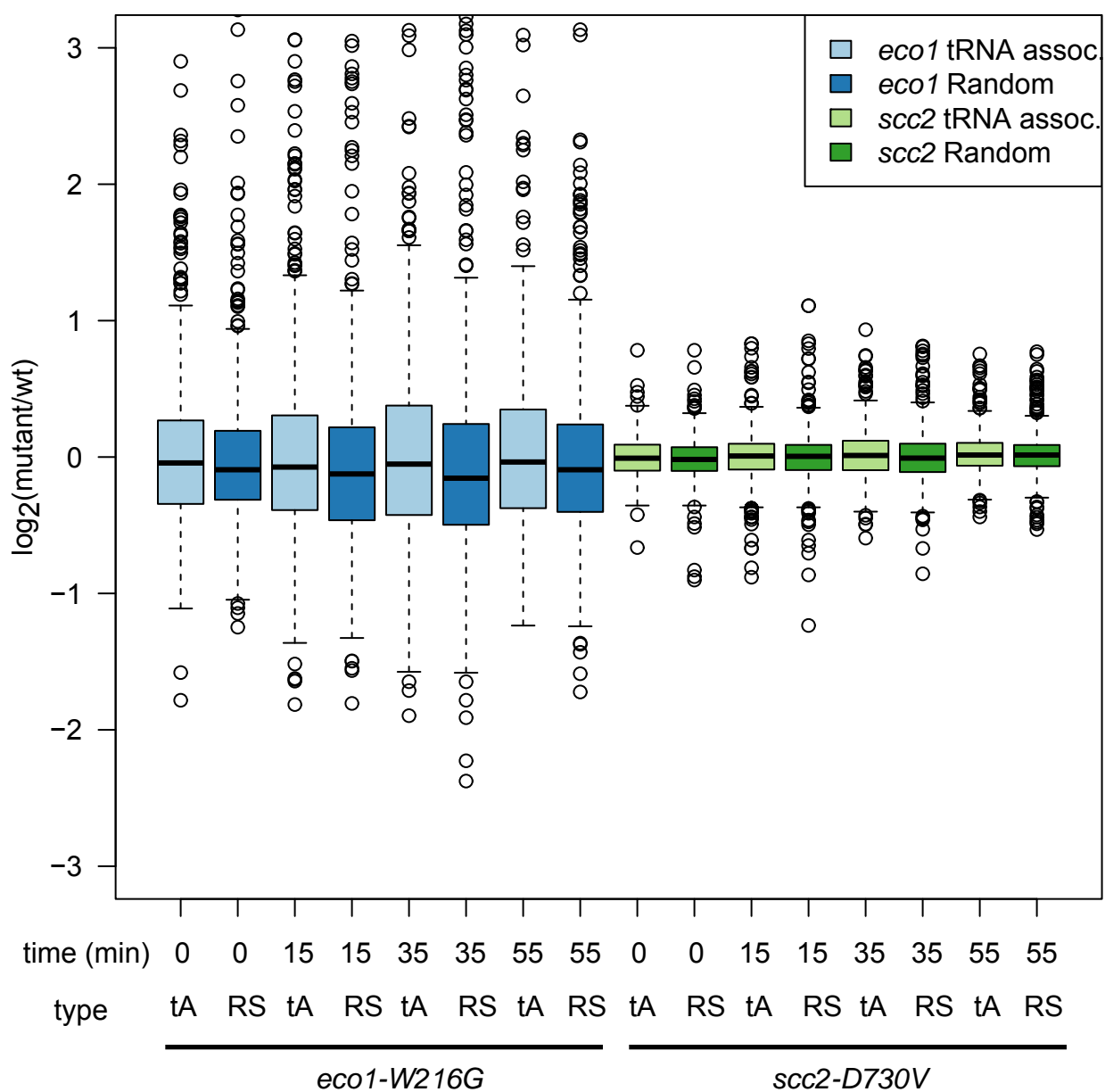

Supplement: Figure S1 — Genes adjacent to tDNAs are not misregulated in cohesin mutants. We examined the expression of genes adjacent to tRNAs in the microarray data set. The coordinates for all yeast tRNA genes were retrieved from Ensembl (299 genes). A script was written to use the Ensembl API and select the nearest gene to the left and to the right of each tRNA. The result was that 35 segments to the left or right of a tRNA gene did not have another gene adjacent within 2 kb, and 67 of the genes returned were another tRNA gene. The remaining gene IDs were mapped against the Affy probe IDs resulting in 418 matches. The mutant/WT expression values for this gene set for each timepoint are mapped in the box plot. In addition, a background set of mutant/WT expression values from 418 randomly chosen genes is also shown. The distribution of expression values from each mutant relative to WT for genes adjacent to a tRNA is shown (tA – tRNA Adjacent). Next to each of these distributions is a set of values from a randomly chosen gene set (RS) from the same dataset. If disruption of the tRNA suppression effect occurs in the mutants, then we would expect to observe an upward shift in the tA distribution relative to the RS set (indicating higher expression of tRNA adjacent genes in the mutant). (PDF) [file pgen.1002749.s001.pdf]

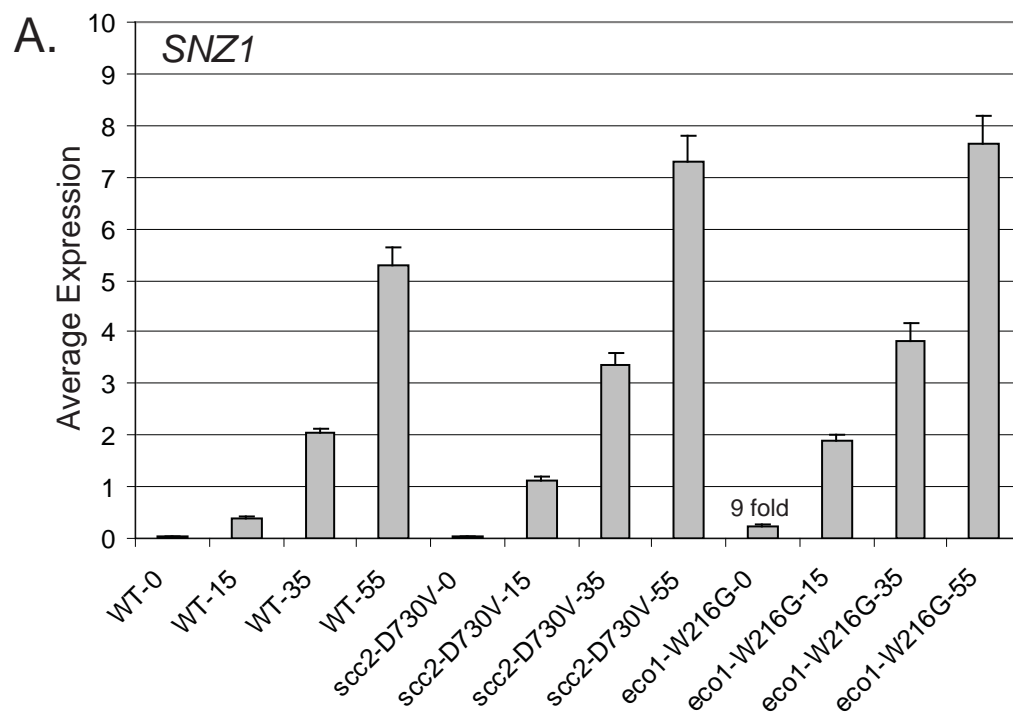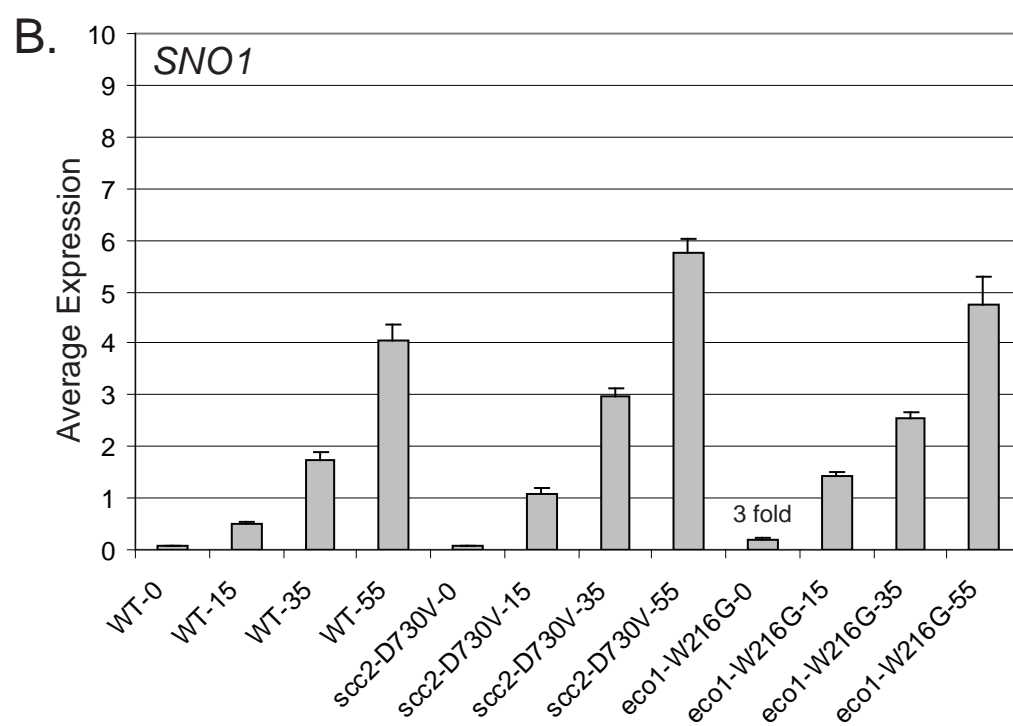

Supplement: Figure S2 — SNO1 and SNZ1 misregulation in cohesin mutants is confirmed by quantitative PCR. RT-qPCR was performed on the RNAs from each timepoint for WT and the two mutants. Gene specific primers for SNZ1 (A) and SNO1 (B) were used and the increase over time was calculated relative to ACT1 and PGK1. Both SNZ1 and SNO1 are more strongly induced in the mutants. The fold change relative to the WT value is shown for the eco1-W216G mutant at time 0 since the scale makes the change difficult to appreciate. Reactions were performed in triplicate and the mean and the standard deviation is shown. (PDF) [file pgen.1002749.s002.pdf]

# SG136

Bose\_Figure S3

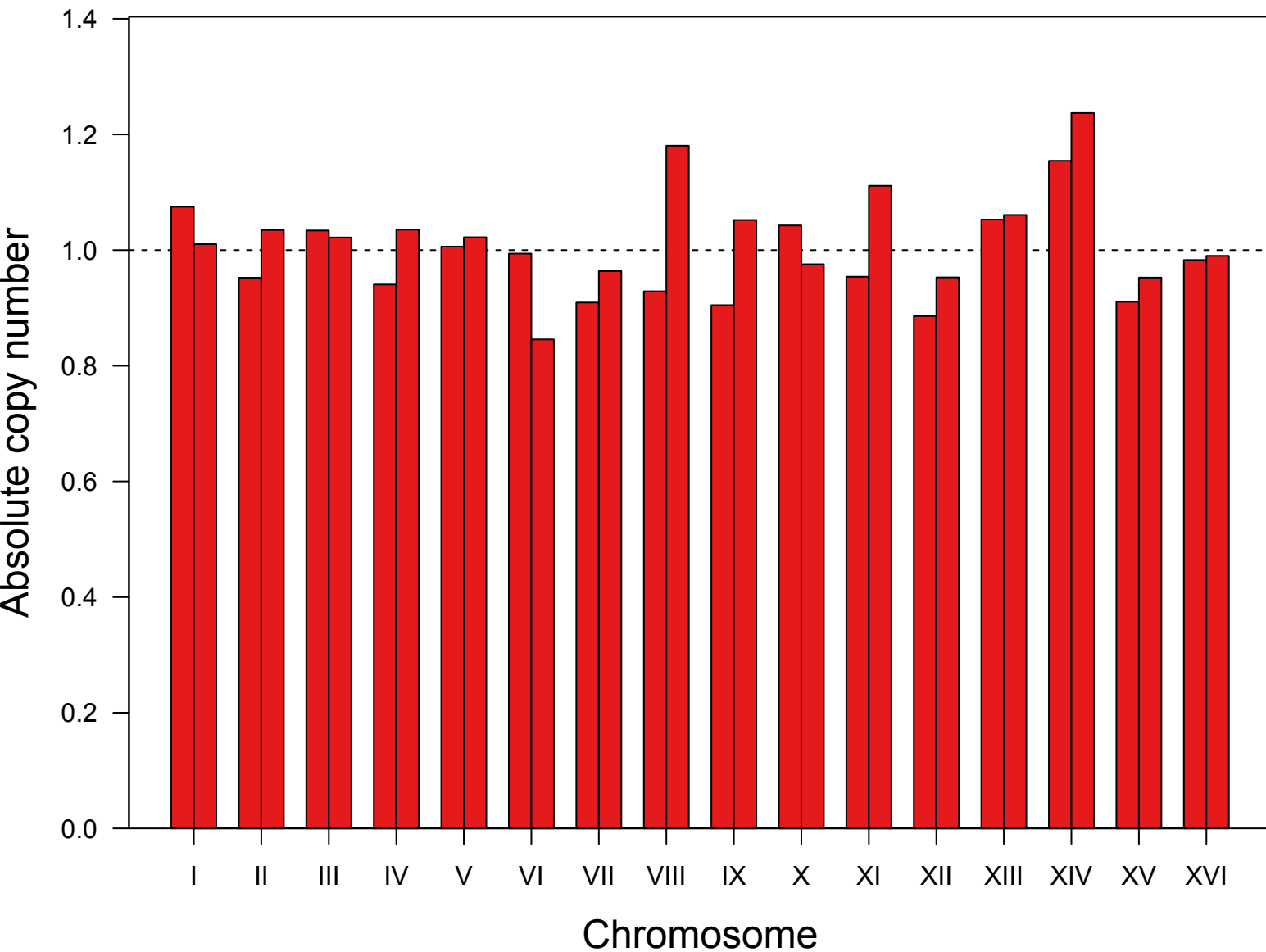

# W303

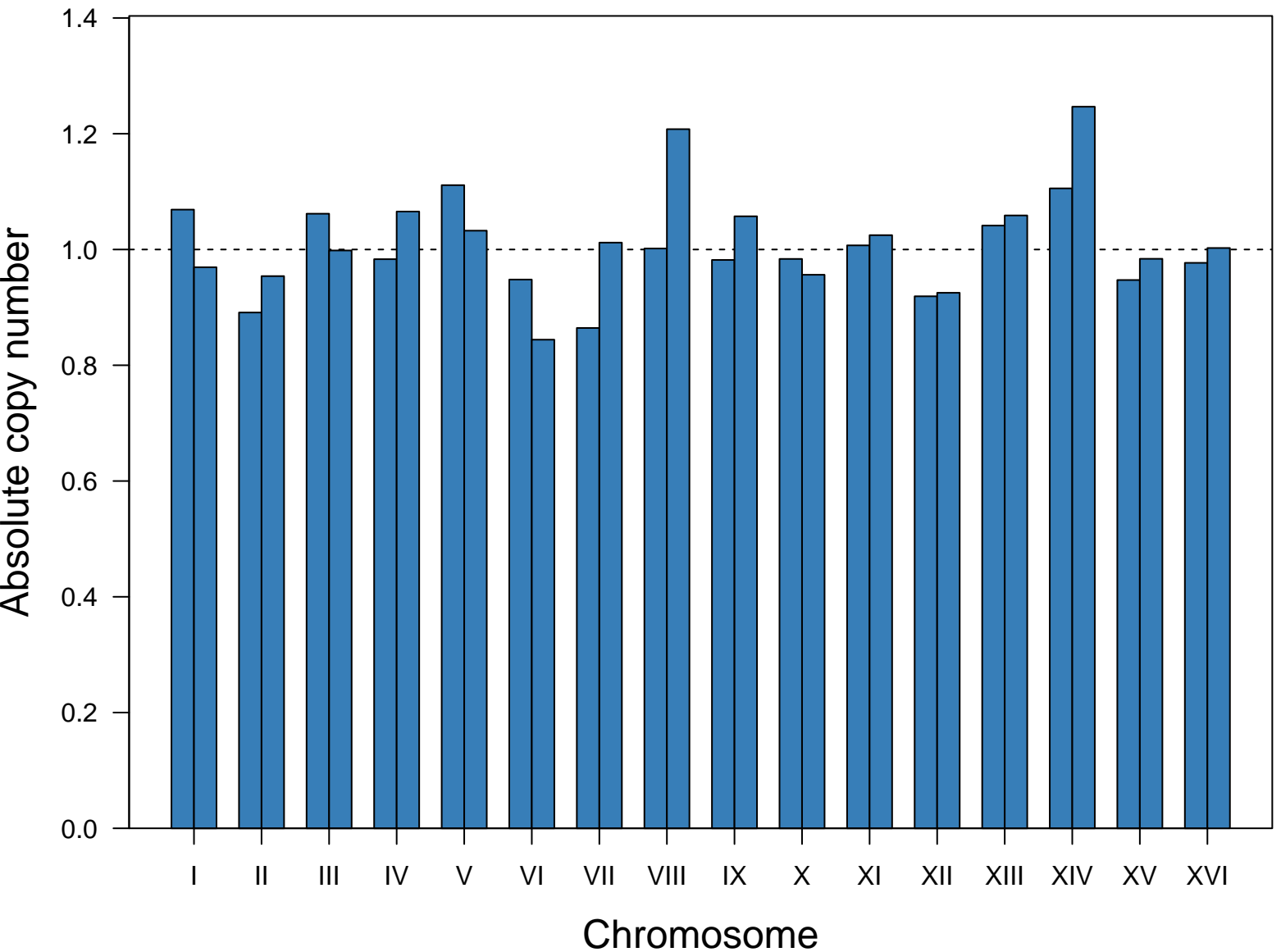

# SG156

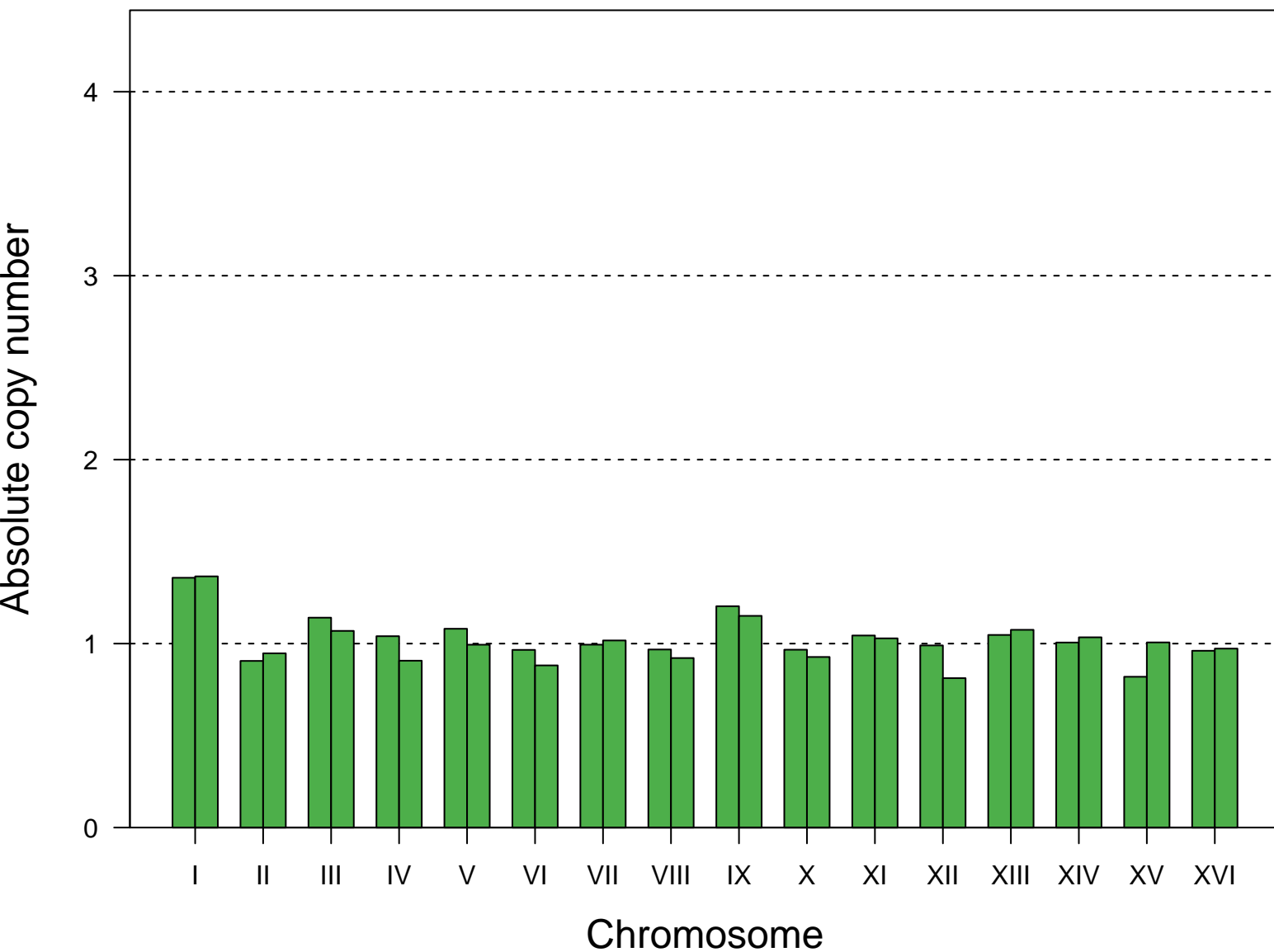

Supplement: Figure S3 — qPCR assay for a sequence on the left and right arm of each chromosome confirms that no aneuploidy is present in either the W303a strain or the smc1-Q843Δ (SG136) and eco1-W216G (SG156) mutants derived from this strain. (PDF) [file pgen.1002749.s003.pdf]

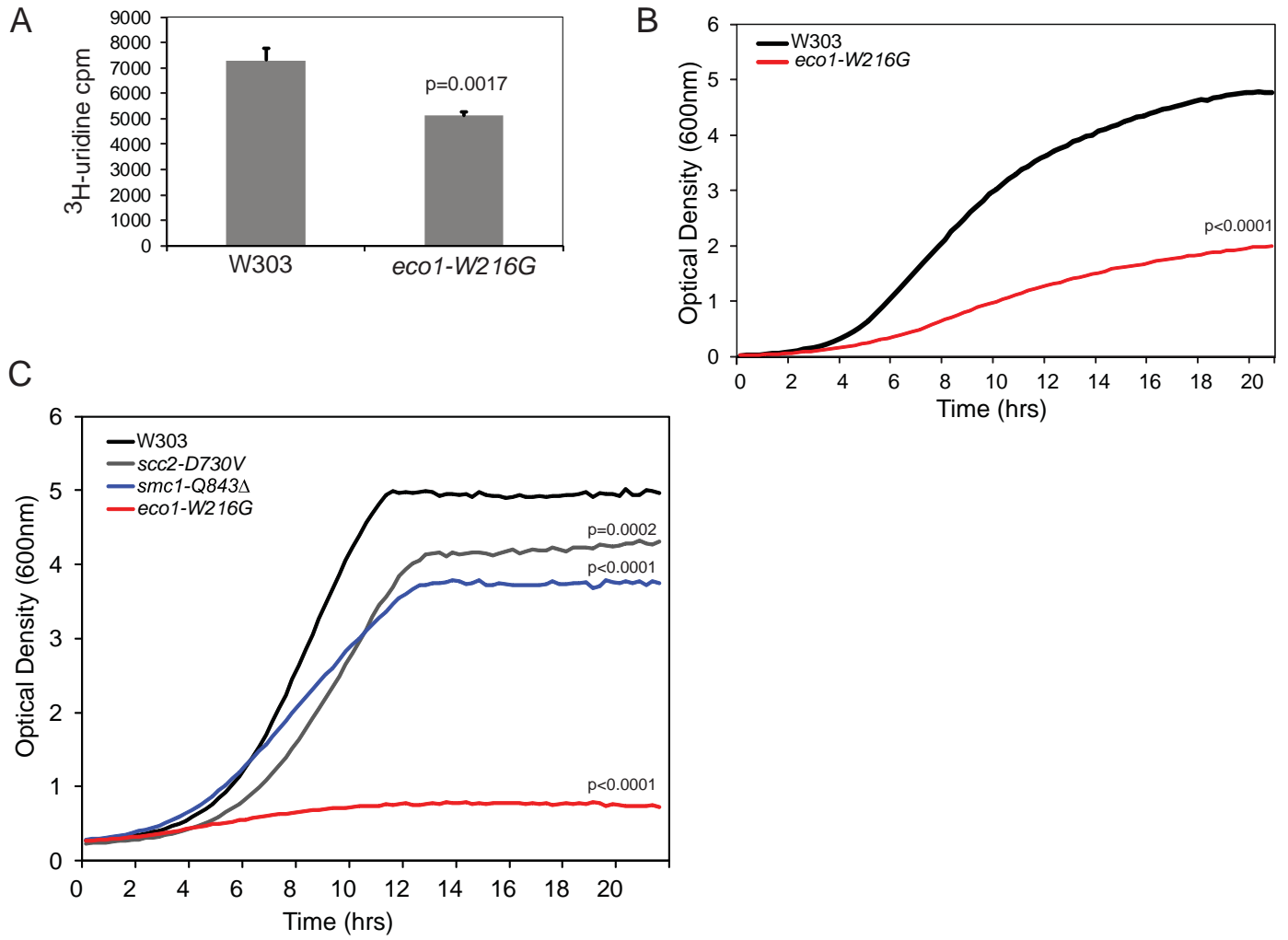

Supplement: Figure S4 — Growth and rRNA labeling. A. RNA was pulse labeled and the incorporation of 3H-uridine was quantified in W303a and eco1-W216G strains as performed in Figure 5. B. The growth of the strains used for (A) in SD-ura at 30°C is shown. C. The growth of the strains indicated is shown in YPD+CSM at 37°C. The growth curve and statistics for B and C were performed as in Figure 3. (PDF) [file pgen.1002749.s004.pdf]

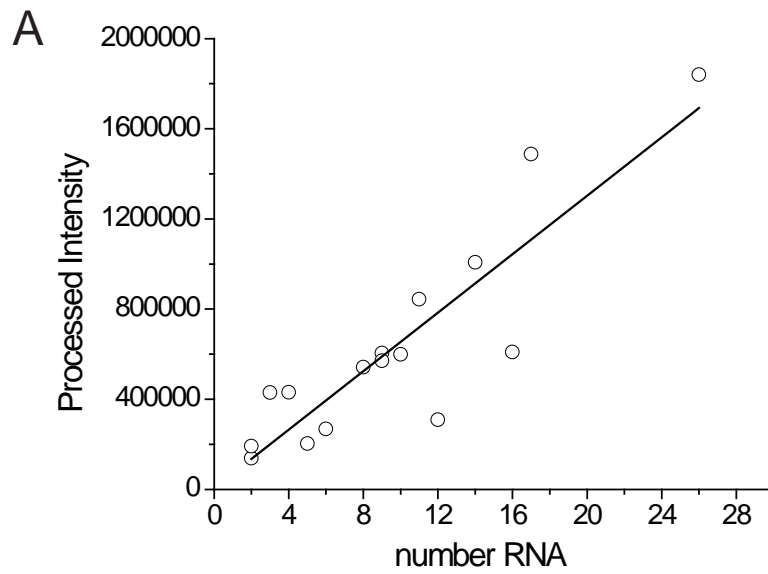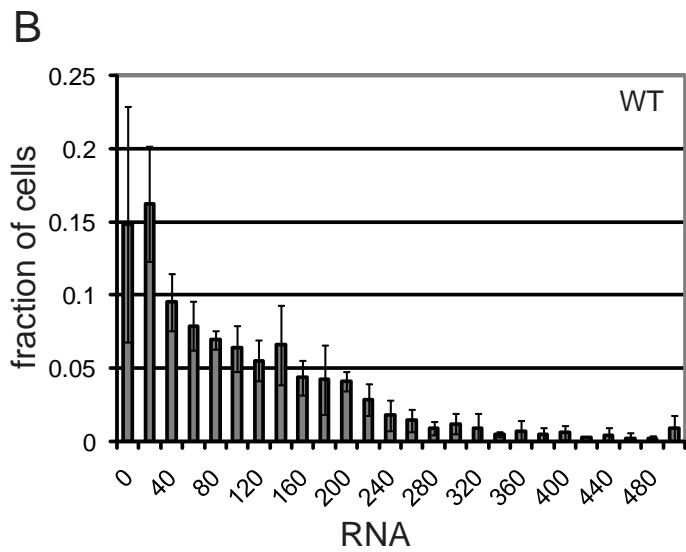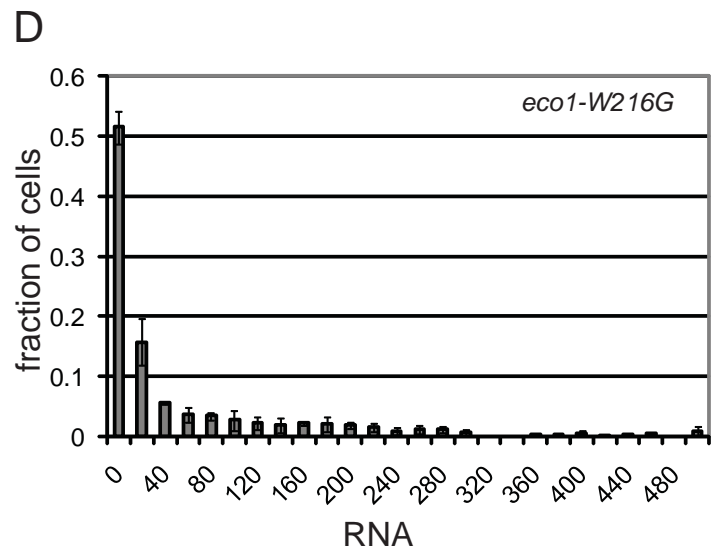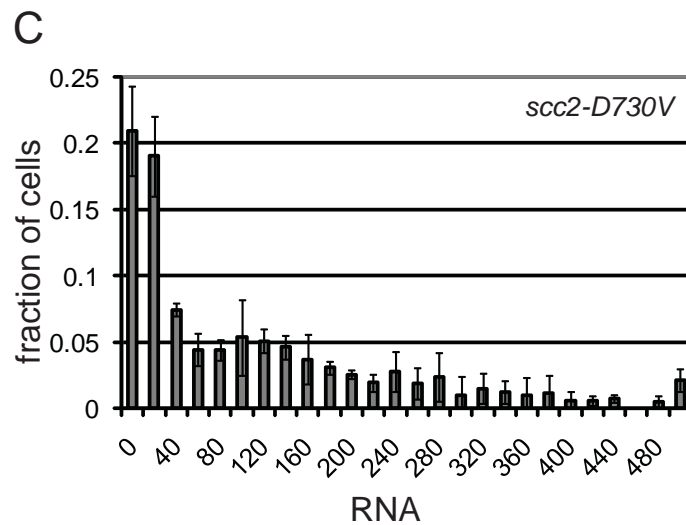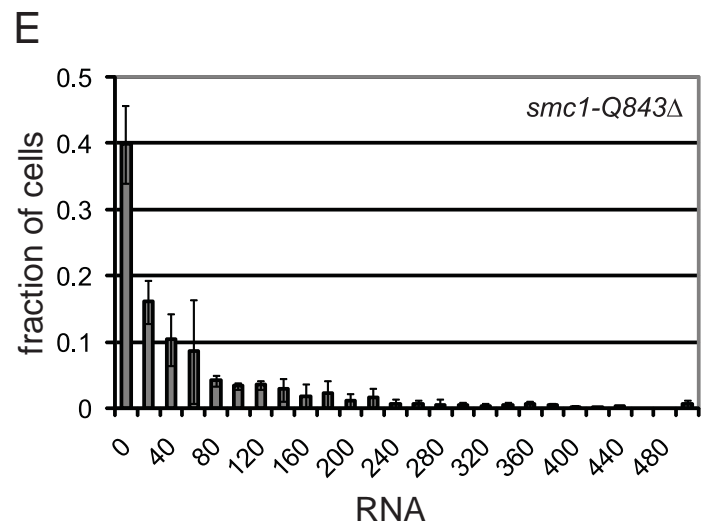

Supplement: Figure S5 — An expanded presentation of the FISH data. A. The standard curve shown was used to determine how fluorescence intensity relates to number of RNAs. The fluorescence for each cell is measured and then binned to show the fraction of the population with each RNA number. The fluorescence for 300 cells from 3 independent cultures for each strain was measured (900 cells total per strain). The error bars indicate the standard deviation. B. WT C. scc2-D730V D. eco1-W216G E. smc1-Q843Δ. (PDF) [file pgen.1002749.s005.pdf]
